# An intersegmental single-cell profile reveals aortic heterogeneity and identifies a novel Malat1^+^ vascular smooth muscle subtype involved in abdominal aortic aneurysm formation

**DOI:** 10.1038/s41392-022-00943-x

**Published:** 2022-04-27

**Authors:** Liwen Yu, Jie Zhang, Amy Gao, Meng Zhang, Zunzhe Wang, Fangpu Yu, Xiaobin Guo, Guohai Su, Yun Zhang, Meng Zhang, Cheng Zhang

**Affiliations:** 1 The Key Laboratory of Cardiovascular Remodeling and Function Research, Chinese Ministry of Education, Chinese National Health Commission and Chinese Academy of Medical Sciences, The State and Shandong Province Joint Key Laboratory of Translational Cardiovascular Medicine, Department of Cardiology, Qilu Hospital, Cheeloo College of Medicine, Shandong University, Jinan, China; 2 Cardiovascular Disease Research Center of Shandong First Medical University, Central Hospital Affiliated to Shandong First Medical University, Jinan, China

**Keywords:** Cell biology, Cardiovascular diseases

## Abstract

The developmental origin, anatomical location, and other factors contribute to aortic heterogeneity in a physiological state. On this basis, vascular diseases occur at different ratios based on position specificity, even with the same risk factor. However, the continuous intersegmental aortic profile has been rarely reported at the single-cell level. To reveal aortic heterogeneity, we identified 15 cell subtypes from five continuous aortic segments by marker genes and functional definitions. The EC1 subtype highly expressed *Vcam1* and *Scarb2* genes in the aortic arch, which were reported to be associated with atherosclerosis. The newly identified Fbn1^+^ fibroblasts were found highly expressed in thoracic segments. More importantly, vascular smooth muscle cells (VSMCs) demonstrated a novel composition in which VSMC 4 marked with the gene *Malat1* were mainly distributed in the abdominal segment. *Malat1* knockout reduced MMPs and inflammatory factor production induced by Ang II in smooth muscle cells, and the Malat1 inhibitor exerted preventive, inhibitory, and reversing effects on AngII-induced abdominal aortic aneurysm (AAA) in vivo revealed by a series of animal experiments. Single-cell analysis of AngII-induced AAA tissues treated with or without the inhibitor further clarified the key role of Malat1^+^VSMC in the occurrence and progression of AAA. In summary, segmental gene expression and cell subtype features in normal aorta associated with different vascular diseases might provide potential therapeutic targets.

## Introduction

The aorta serves as an elastic pipeline transforming the heart output with high pressure into flow with moderate fluctuations.^1^ The unique embryological basis^2–4^ and hemodynamic features^5^ of the aorta create its microenvironment and heterogeneity, in which aortic segments show region-specific diversity. Under pathological conditions, the atherosclerotic lesions at the aortic arch appear earlier and more severe than at other positions.^6^ Abdominal aortic aneurysm is one of the most severe vascular diseases with a high incidence and mortality but low control rate.^7^ The most typical and generally accepted animal model of AAA is the AngII-induced model, which mimics most human AAA features.^8^ However, more than 95% of this model with AngII infusion throughout the body can only cause aneurysms in the abdominal aorta (Supplementary Fig. S2a). These phenomena suggest that the intrinsic gene expression and cell subtypes in consecutive healthy vessel segments are discrepant and that different segments perform with different sensitivity to specific risk factors.

In previous studies, when constructing the single-cell landscape of all major organs in humans or mice,^9,10^ the aorta was often ignored as a small part of the complicated organ system. The primary single-cell analysis of the normal aorta provided a basic transcriptome, in which Vcam1^+^ endothelial cells were well documented in 2019 by Kalluri et al. showing their anatomical location in the aortic root.^11^ Most studies concentrated on endothelial cells^12,13^ and neglected the main structural cell type, VSMCs. Although recent studies of aortic diseases performed by single-cell RNA sequencing (scRNA-seq) have emerged, they ignored the different incidence of positions.^14,15^ Notably, regarding regional heterogeneity, the single-cell atlas of the aorta has rarely been reported in normal conditions.

To explain the aortic spatial heterogeneity, we used scRNA-seq to generate a comprehensive profile of the full-length aortic segments. This study showed that the EC1 subtype highly expressed *Vcam1 and Scarb2* genes in the aortic arch, which are reported to be associated with atherosclerosis. Two fibroblast subclusters showed specific differentially expressed genes with opposite functions in extracellular matrix metabolism, in which new Fibrillin 1^+^ (Fbn1^+^) cells mainly colocalized with fibroblast 1 and highly expressed in thoracic segments. A novel compositional system of VSMC subpopulations was identified instead of the traditional contractile and secretory types involved in pathological vascular remodeling.^16^ A special VSMC subtype marked with Malat1 was central in the healthy abdominal aorta.

Previous studies of *Malat1* have focused on cancer research, particularly with respect to breast cancer invasion and metastasis.^17^ In cardiovascular studies, it has been reported that *Malat1* might play roles in stenotic vascular diseases and intracranial aneurysm.^18,19^ Until now, none of the existing studies had revealed the role of *Malat1* in classic AngII-induced AAA model. The present study provides a new perception and research direction for aortic diseases from the perspective of a normal segmental aortic transcriptome at the single-cell level. Through single-cell sequencing technology and molecular biology experiments, we revealed that Malat1^+^ VSMCs, with a high degree of specificity in the normal abdominal aorta segment, might be an important mediator of AAA susceptibility induced by AngII. Then, based on a series of animal model experiments, we explored the preventive, inhibitory, and reversing effect of a *Malat1* inhibitor, which provided a novel therapeutic strategy for clinical AAA treatment. Finally, we verified these conclusions once again by single-cell analysis to confirm the dominant status of Malat1^+^ VSMCs and evaluated the inhibitor effect at the single-cell level.

## Results

### Single-cell transcriptome atlas of the aorta by simulating spatial distribution

**Fig. 1:**
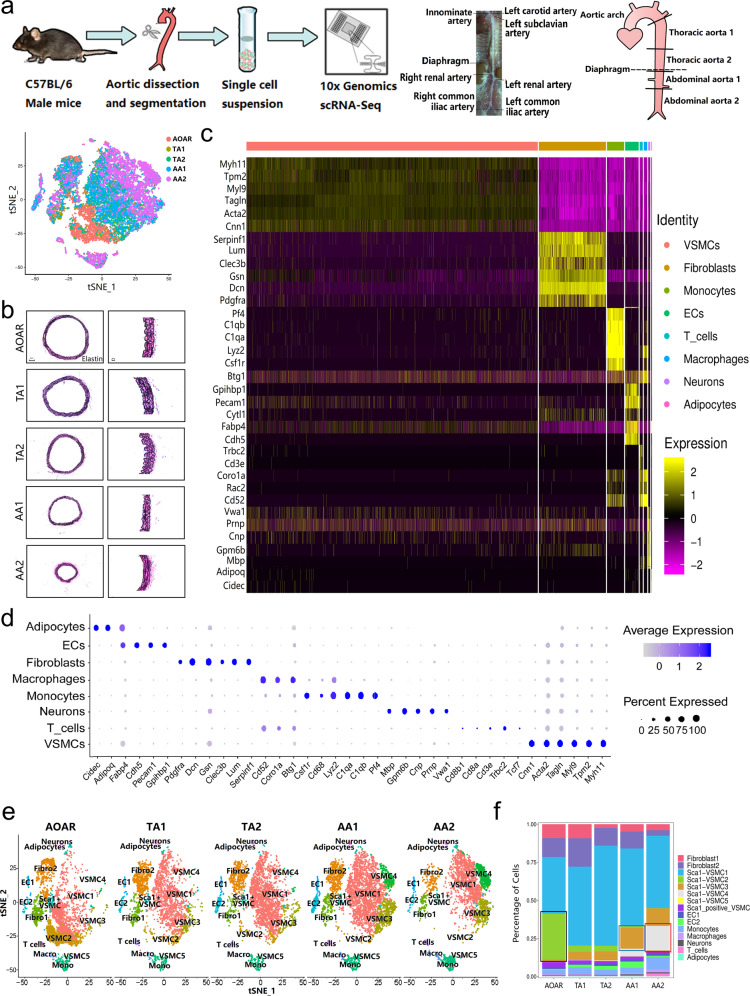
Profiling of different aortic segments using scRNA-seq. **a** Experimental procedure for five consecutive aortic segments. Full-length aortas were separated from five C57BL/6 male mice at 12 weeks and dissected into five segments based on the anatomical landmarks. Different aortic segments were digested with the same dissociation program to obtain single-cell suspensions for sequencing with 10X Genomics. **b** Elastin staining of five segments showed the complete structure of the aorta with decreased diameter. **c** The heatmap showed top marker genes to identify all aortic cells into eight types. The order of marker genes was in accordance with the order of cell types. **d** A dot plot indicated the relative expression of marker genes in the distinct cell population. The dot size reflects the percentage of cells expressing the selected gene in each population. **e** The distribution of all subtypes in different segments was manifested by the t-SNE plot. **f** The proportion of special subclusters showed significant variation among five aortic sections, especially VSMC 2, 3, and 4. EC endothelial cell, VSMC vascular smooth muscle cell, Fibro fibroblast, Macro macrophage, Mono monocyte

To reveal the heterogeneity of the aorta originating from the aortic root to the arteria iliaca communis, we applied scRNA-seq to derive a detailed transcriptome of aortic cells from five male wild-type C57BL/6 mice (Fig. 1a). Considering the anatomical landmark differences between humans and mice, we divided the aorta into five segments, aortic arch (AOAR), thoracic aorta 1 (TA1), thoracic aorta 2 (TA2), abdominal aorta 1 (AA1), and abdominal aorta 2 (AA2), all with complete and clear elastin structures (Fig. 1a, b). After digestion with enzymes, five cell suspensions with stringent quality control (Supplementary Fig. S1) were sequenced using a 10X genomics system. We obtained 26,257 cells in all and 5250 cells of each segment on average, consistently distributed in the t-SNE plot (Fig. 1a). All aortic cells were sequenced at a high sequencing depth (60,470 reads per cell), and nearly 1600 genes were detected per cell. We obtained 17 clusters of cells from five aortic segments (Supplementary Fig. S2b).

All aortic cells were defined by eight types shown by the heatmap and the dot plot (Fig. 1c, d). The top markers could be identified based on genome alignment (Supplementary Table S2) with CellRanger involving classic marker genes,^11^ such as *Pecam1*, *Cdh5* for ECs, *Myh11*, *Tagln* for VSMCs, *Pdgfra*, *Gsn* for fibroblasts, *C1qa*, *Pf4* for monocytes, and *Cd3e*, *Trbc2* for T cells, among others. VSMCs were the main component of the aortic wall in the structure (Supplementary Fig. 2b, c). The distribution ratio of different subtypes varied from segment to segment, particularly the VSMCs. Three specific subtypes of VSMCs demonstrated a continuous increasing or decreasing trend in terms of the ratio and number. VSMC 2 gathered at the AOAR section, whereas VSMCs 3 and 4 were predominant on the abdominal part (Fig. 1e, f and Supplementary Fig. S2d).

### ECs and fibroblasts showed segmental differentially expressed genes but no distribution difference

**Fig. 2:**
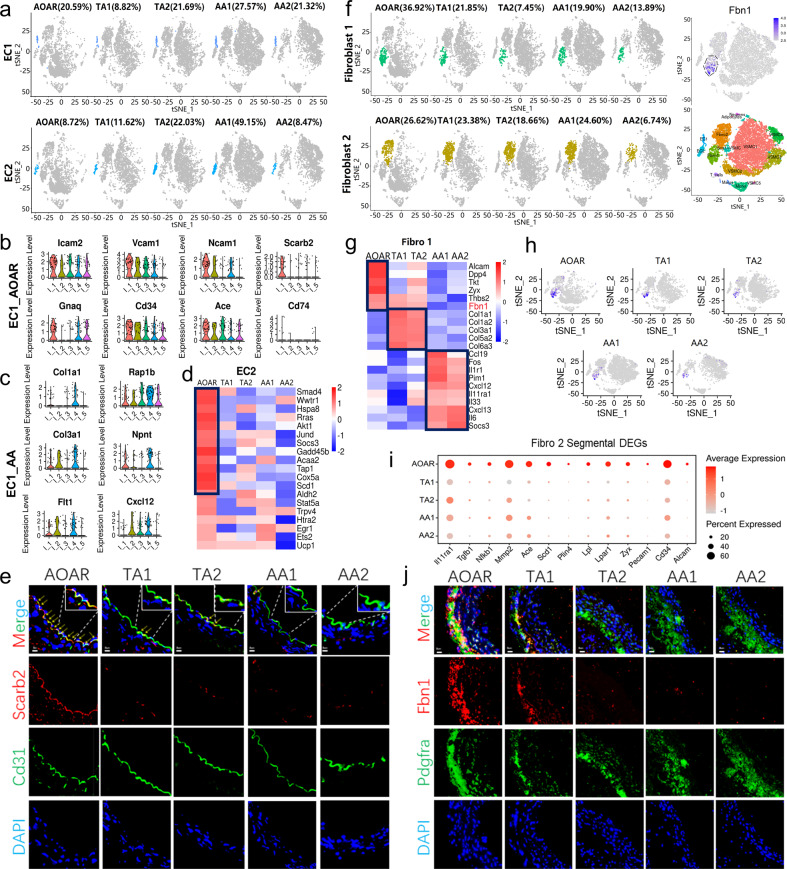
Segmental DEGs in EC and fibroblast subtypes without differences in the ratio of distribution. **a** The t-SNE suggested the proportion of EC subtypes among five segments without significant differences (*P* > 0.05). **b**, **c** The enriched genes of EC1 with high expression mainly focused on the AOAR and AA1 segments, including focal adhesion genes, such as *Vcam1* and *Icam2* in AOAR and ECM metabolism genes, such as *Col3a1*. I_1, I_2, I_3, I_4, and I_5 in X axes represented AOAR, TA2, TA2, AA1, and AA2, respectively. **d** The highly expressed genes of EC 2 were only enriched in AOAR including *Smad4* and *Scd1*, among others. **e** Verification of *Scarb2* expression in ECs across five segments showed that the expression of *Scarb2* was enriched in the AOAR portion by immunofluorescent localization. The arrows and the partially enlarged images were used to indicate the expression of *Scarb2* in ECs. Scale bar = 5 μm. **f** The distribution of two fibroblast subtypes across five segments was shown by t-SNE without a statistical difference (*P* > 0.05). The feature plots showed that the Fbn1^+^ cells colocalized with fibroblast 1 when compared to that with all the cell types. **g** Segmental gene heatmap of Fibro1 with a significant spatial difference. The collagen-related genes *Col1a1* and *Col3a1* were highly expressed in TA segments, whereas inflammation factors *Il1r1* and *Il6* showed higher expression in AA segments. **h** Regarding spatial distribution, Fbn1^+^ cells were mainly distributed in the thoracic part of the aorta, particularly in the first section, the AOAR. **i** The Fibro 2 expressed specific differential genes mainly focused in the AOAR portion, such as *Tgfb1* and *Mmp2*. **J** Immunofluorescence staining showed that the expression of *Fbn1* was high in the thoracic aorta, particularly in the aortic arch. Scale bar = 5 μm. Each experiment was repeated independently for a minimum of three times

As the first barrier of the aortic lumen, ECs are inseparable from substance exchange^20^ and adjacent to injury sites during the initial damage response.^21^ ECs were identified as EC1 and 2 based on relatively highly expressed genes, such as *Vcam1* and *Ace* for EC1, *Cd36,* and *Lpl* for EC 2 (Supplementary Fig. S3a). Functional enrichment analysis showed that EC1 was enriched in hemodynamic pathways reflected by the marker genes *Bmx* and *Ctsh* (Supplementary Fig. S3b, c). In addition to lipid metabolism, EC 2 manifested more activity in the series of Ras signaling pathways (Supplementary Fig. S3c). However, the distribution of EC1 and 2 showed no difference among the five segments (Fig. 2a).

Regarding spatial gene expression, *Vcam1* and *Scarb2* in EC1 were highly expressed in the AOAR segment (Fig. 2b). The collagen-associated genes, including *Col1a1*, were enriched in the AA1 segment (Fig. 2c). The differentially expressed genes (DEGs) also reflected the trend in pathways enriched in cell adhesion and antigen processing and presentation in the AOAR segment (Supplementary Fig. S3d). In EC 2, the MAPK signaling-related genes, including *Smad4* and *Akt1*, were mainly enriched in the AOAR segment (Fig. 2d). KEGG enrichment analysis of DEGs showed that the PPAR signaling pathway was upregulated, focusing on the AOAR segment (Supplementary Fig. S3d). The expression of *Vcam1* and *Scarb2* in EC1 across five segments was verified by immunofluorescence localization and was highly expressed in the AOAR segment, consistent with the single-cell data (Fig. 2e and Supplementary Fig. S3e).

Fibroblasts play an irreplaceable role in vascular remodeling and contribute to neointimal formation.^22,23^ There was no significant difference in the distribution of the two fibroblast subtypes across the five segments (Fig. 2f). The fibroblast subtypes were defined as fibroblast 1 (*Clec3b,*^24^ *Serping1*) and fibroblast 2 (*Fmod*, *Comp*) based on the highly expressed genes (Supplementary Fig. S4a). The gene *Pi16*^25^ highly expressed in fibroblast 1 was related to the negative regulation of tissue damage repairment.^26^ In contrast, the marker gene *Fmod* in fibroblast 2 affected the rate of fibril formation^27^ (Supplementary Fig. S4b). In conclusion, the gene expression of fibroblast 1 was more inhibitory of matrix synthesis than type 2. Moreover, KEGG analysis revealed that fibroblast 1 showed gene enrichment in TNF signaling pathways, whereas fibroblast 2 mainly participated in actin skeleton regulation (Supplementary Fig. S4c).

Fibrillin 1 participates in critical biomechanical processes including anchoring, connecting, and maintaining tissues.^28^ A previous study highlighted the systemic action of *Fbn1* based on Fbn1^C1039G/+^ mutant mice.^29^ We detected that *Fbn1* was specifically expressed in fibroblast 1 (cluster 7) by comparing all cell types or fibroblasts separately (Fig. 2f and Supplementary Fig. S4d). Although there were no differences in the distribution of fibroblast subtypes, the segmental difference in gene expression was still significant. For example, the expression of *IL6* and *Cxcl12* in fibroblast 1 was highly expressed in the AA segments (Fig. 2g). The genetic differences in fibroblast 2 were mainly reflected in the AOAR part (Fig. 2i). Moreover, Fbn1^+^ cells were mainly distributed in the thoracic aorta, particularly in the AOAR segment (Fig. 2h and Supplementary Fig. S4e). Detection of *Fbn1* by immunofluorescence colocalization was also verified at a high level in the thoracic aorta (Fig. 2j).

### Identification of a novel constitution system of VSMC subpopulations

**Fig. 3:**
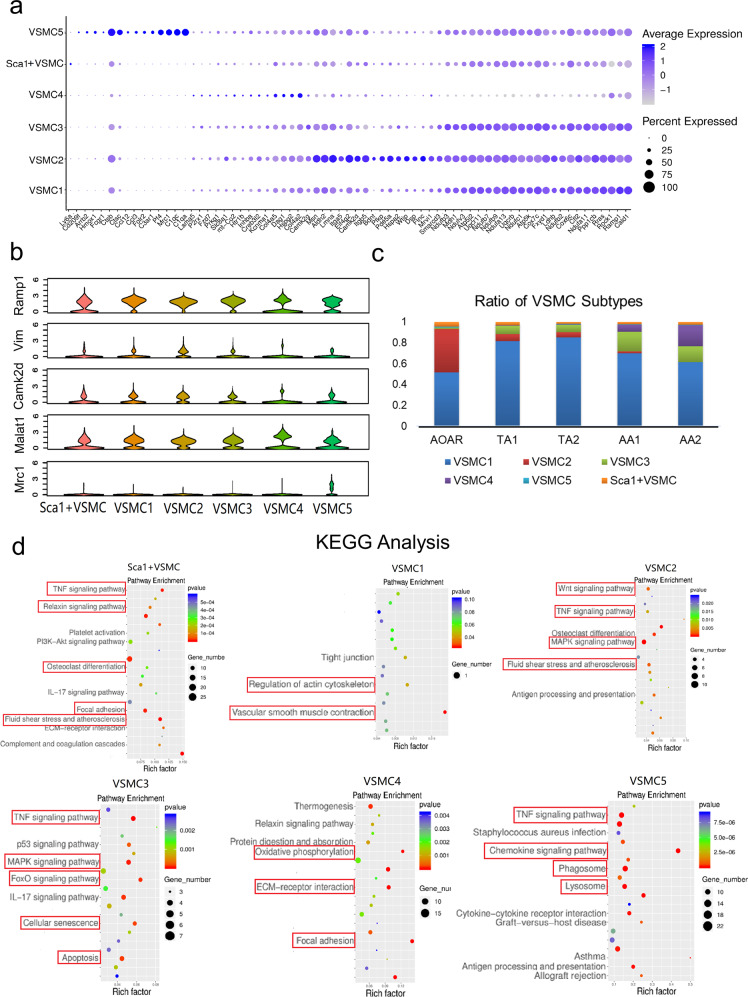
System of vascular smooth muscle cell (VSMC) subtypes performing their own functions. **a** Vascular smooth muscle cells could be subdivided into six subpopulations marked with different genes shown by the dot plot. The VSMC subtypes featured a series of correlative marker genes involving a similar expression. **b** For each subtype, one representative gene was chosen as a potential detection target as follows: *Ramp1* for VSMC 1, *Vim* for VSMC 2, *Camk2d* for VSMC 3, *Malat1* for VSMC 4, *Mrc1* for VSMC 5. **c** The proportion of each VSMC subtype in all smooth muscle cells across different segments is displayed by bar graphs. VSMC 2 showed a declining trend, whereas VSMC 3 and 4 presented with an increasing trend, consistent with the phenomenon across all cell types. **d** KEGG analysis confirmed the characteristic enriched pathways of the six VSMC subtypes

In traditional research, VSMCs are divided into two types, contractile and synthetic, during the progression of vascular diseases.^30,31^ According to comparisons of gene expression, such as *Sm22α* for the contractile type and *Opn* for the synthetic type, the VSMCs were almost all contractile, with few synthetic VSMCs in the healthy mouse aorta. Therefore, in this study, we created a more detailed classification of the healthy state and eventually identified six distinct subtypes, including VSMCs 1–5 and Sca1^+^VSMCs. The identification of VSMCs 1–5 was defined by the specific DEGs, namely *Ramp1* for VSMC 1 (cluster 0, 1, 2, 4), *Vim* for VSMC 2 (cluster 5), *Camk2d* for VSMC 3 (cluster 6), *Malat1* for VSMC 4 (cluster 8), and *Mrc1* for VSMC 5 (cluster 16) (Supplementary Fig. S2b and Fig. 3a, b).

The progenitor, Sca1^+^VSMCs, existed in a specific small number, maintaining stem cell-like properties^32^ (Supplementary Fig. S5a). VSMC 1 was the most abundant in quantity among the six subtypes (Fig. 3c). The enrichment analysis proved that VSMC 1 was the most differentiated subtype of VSMC and highly enriched in pathways associated with VSMC contraction phenotype (Fig. 3d and Supplementary Fig. S5b). Both Sca1^+^VSMCs and VSMC 1 were distributed evenly across the five segments. The smallest subtype was VSMC 5 with no distribution difference (Supplementary Fig. S6a), but it exhibited similar immune features to monocytes (Supplementary Fig. S6b–g).

### Analysis of three distinct gradient-distributed VSMC subtypes by branching gene expression trajectory

**Fig. 4:**
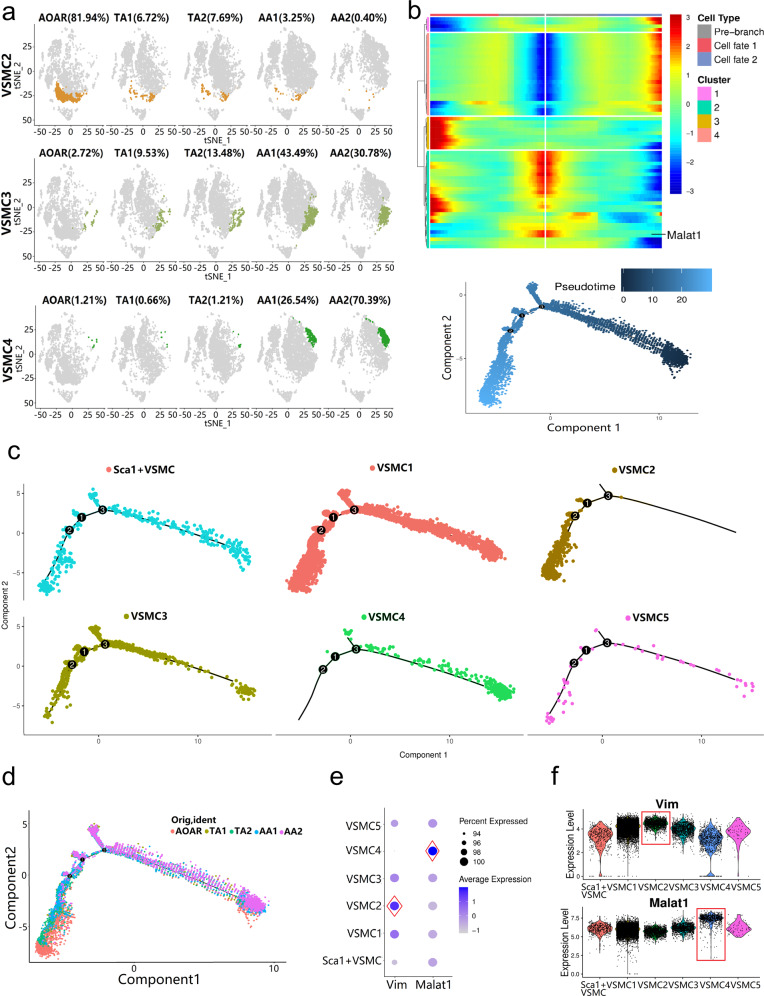
New special VSMC subtypes with segment distribution differences revealed by gene characteristics and branching gene expression trajectory analysis. **a** The gradient of spatial distribution occurred among three particular VSMC subtypes, VSMC 2, 3, and 4, which was visualized by the t-SNE plot. **b** Branching gene expression trajectory analysis was performed based on dependent genes, including *Malat1*. The pseudotime direction of cell differentiation is from right to left. **c** Different subpopulations appeared in diverse evolutionary directions. VSMC 2 and 4 developed into two opposite differentiation directions. **d** The five segments resulted in the direction dominated by subtype differentiation, particularly the difference between thoracic and abdominal segments. The differentiation direction of AA was consistent with the VSMC 4. **e** The expression of genes *Vim* and *Malat1* in VSMC 2 and 4 separately was relatively higher than that in the other subtypes. **f** Cells belonging to VSMC 2 and 4 converged on a high expression level of markers in the violin plots (*P* < 0.05 by one-way ANOVA)

The remaining three types of VSMCs showed gradient changes in spatial distribution across the five segments. The number of VSMC 2 decreased from the AOAR to the AA2 segment and was concentrated in the AOAR section (81.94%) (Fig. 4a). In contrast, VSMC 4 showed an increasing trend and was mainly distributed in the abdominal region, particularly in the AA2 section (70.39%). The transition state VSMC 3 had a smaller amplitude and gradually increased from the thoracic to the abdominal part. According to enrichment analysis, the three subtypes of VSMCs had similarities and overlapping functions (Fig. 3d and Supplementary Fig. S5b). VSMC 4 showed enriched genes in pathways related to ECM–receptor interactions and extracellular matrix organization.

To obtain independent evidence for the putative topography of the various VSMC subclusters, we performed pseudotime analysis to predict the differentiation trajectory of VSMC subclusters. There were three different cell fates of VSMCs (Fig. 4b). Then we matched the VSMCs with different fates of cells from subtypes and segments. Among the six subtypes, VSMC 2 and 4 developed into two opposite differentiation directions (Fig. 4c). In five segments, the abdominal portion (AA) tended to be the branch where VSMC 4 was located, whereas the thoracic part (AOAR, TA) was directed consistently by VSMC 2 (Fig. 4d). Then the marker genes *Vim* and *Malat1* were identified with high expression in VSMC 2 and 4, respectively (Fig. 4e). The basic level of *Malat1* in all VSMCs was significant. However, the expression in VSMC 4 remained significantly higher than that in the other types (*P* < 0.05, one-way ANOVA analysis; Fig. 4f).

### Verification of VSMC 4 marked with *Malat1* in both normal and AAA tissue

**Fig. 5:**
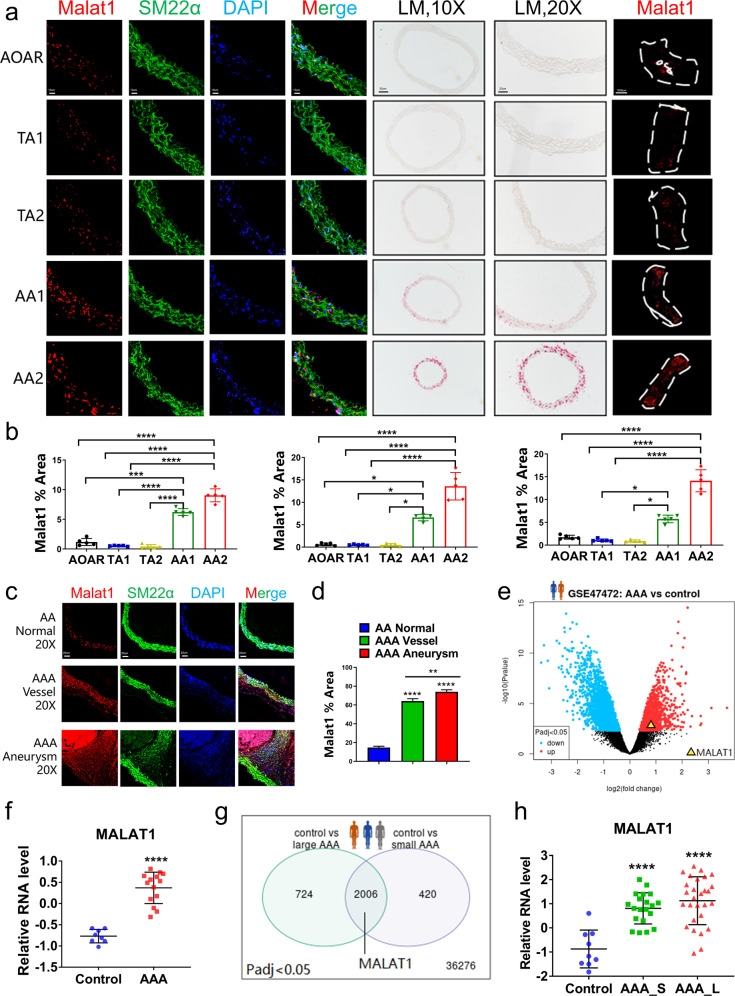
Detection of specific marker *Malat1* of Sca1^−^VSMC 4 in different healthy aortic portions and AAA tissue. **a** *Malat1*, detected by fluorescence in situ hybridization (FISH) or whole-mount in situ hybridization (WISH), at both the transverse section and tissue fragments verified the distribution of VSMC 4. Scale bars = 10 μm, 50 μm, 20 μm, and 1000 μm, respectively. **b** The percentage of the Malat1-positive area in the AA portion either AA1 or AA2, was significantly higher than that in the others. *n* = 5 for each group. **c** Detection of the expression level of *Malat1* in AAA, including both vessel and aneurysm parts, compared with that in the AA segment at baseline. Scale bar = 20 μm. **d** The percentage of the Malat1-positive area in the aneurysm was much higher than that in the normal abdominal aorta. *n* = 5 for each group. **e** Analysis of the human AAA database 1 (GSE47472, *n* = 14 patients in the AAA group, *n* = 8 donors in the control group) showed that MALAT1 was upregulated in human AAA tissue. **f** The relative RNA expression level was compared between AAA and control groups, in which MALAT1 was upregulated in the AAA group with a significant difference. **g** An analysis of human AAA database 1 (GSE57691, *n* = 28 patients in large AAA group, *n* = 20 patients in small AAA group, *n* = 9 donors in control group) provided evidence of differential expression of MALAT1. **h** The relative RNA expression level in AAA_L and AAA_S was significantly higher than that in the control group. AAA_L, large AAA. AAA_S, small AAA. Data were presented as mean ± SEM and normal distributions were tested by the Shapiro–Wilk method, which showed that all data were normally distributed. One-way ANOVA followed by Tukey post hoc test was used for (**b**, **d**, **h**). The student’s *t* test was used for (**f**). *P < 0.05, **P < 0.01, ***P < 0.001, ****P < 0.0001. Each experiment was repeated independently for a minimum of three times

To verify the quantity of VSMC 4 marked with *Malat1* in five segments, we detected this marker by FISH in transverse sections with colocalization with the VSMC marker *Sm22α* (Fig. 5a). Under fluorescent or bright-field conditions, the density of *Malat1* staining in the transverse sections of AA segments was much higher than that in thoracic segments (*P* < 0.0001; Fig. 5b), which strongly supported the results of single-cell sequencing. Endothelial cells of each aortic fragment were scraped completely before experiments. Thereafter, the fragments subjected to whole-mount in situ hybridization were also used to detect *Malat1* with high expression at AA segments with a higher threshold control (Fig. 5a, b).

In the AngII-induced mouse model, we detected the expression of *Malat1* in AAA, including in both vessel and aneurysm parts. FISH results suggested that the level of *Malat1* in AAA tissue was much higher than the physiological baseline in the AA segment, particularly in the aneurysm part (*P* < 0.01, one-way ANOVA; Fig. 5c, d). To determine whether similar *Malat1* expression occurs in human AAA, we analyzed RNA-seq data from two public databases online^33,34^ (*n* = 22 patients, *n* = 57 of 68 patients). Analysis of the AAA (*n* = 14) and control group (*n* = 8) in the first database showed upregulated MALAT1 by the volcano plot (Fig. 5e). The relative expression level of MALAT1 in the AAA group was significantly higher than that in the healthy donor group (*P* < 0.0001; Fig. 5f). The patients from another database were divided into three groups, control (*n* = 9), large AAA (*n* = 22), and small AAA (*n* = 20). The results also provided evidence of the high level of MALAT1 expression in human AAA tissue (*P* < 0.0001), even in the small-size AAA (Fig. 5g, h). In addition, a previous study of noncoding RNA sequencing in aortic aneurysm diseases also identified a few upregulated lncRNAs as potential targets, indicating the status of MALAT1.^35^

### Inhibiting *Malat1* remarkably protected VSMCs from AngII stimulation in vitro

**Fig. 6:**
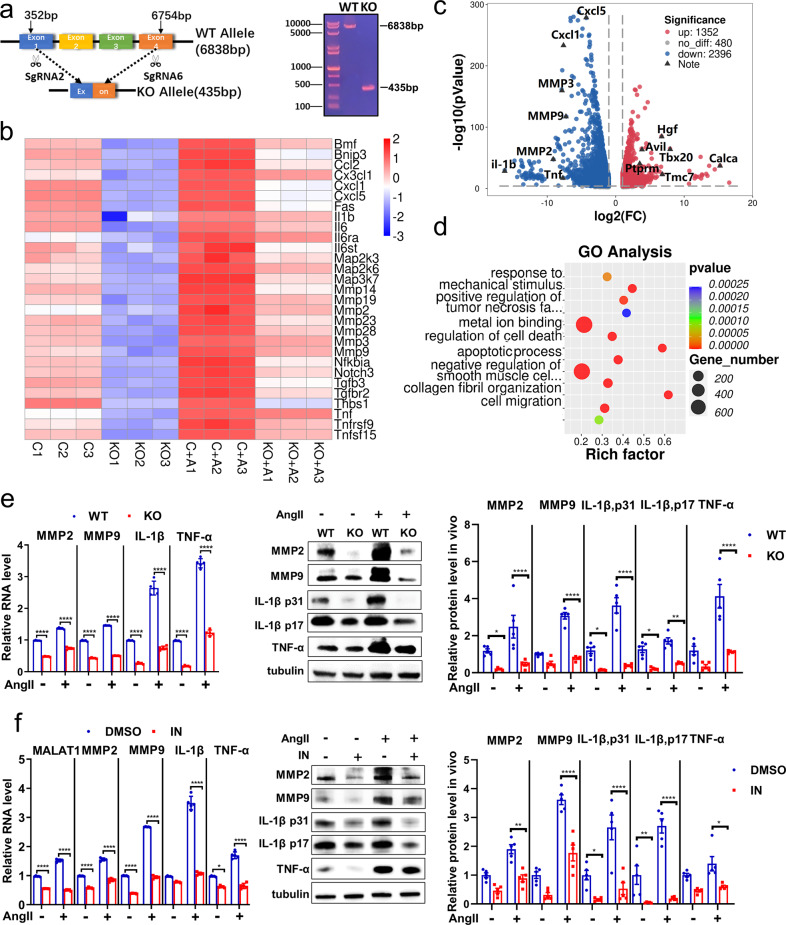
Inhibiting *Malat1* can remarkably protect smooth muscle cells from AngII stimulation in vitro. **a** The monoclonal MOVAS cells with stable knockout of the *Malat1* gene using the CRISPR/Cas9 system via lentiviral transfection were screened. **b** RNA transcriptome sequencing of Malat1^−/−^ and WT MOVAS cells at baseline and with AngII stimulation. A heatmap of downregulated genes showed relative expression (*z* score) levels of abundantly and differentially expressed genes. **c** The fold change of all genes with a significant difference was presented in the volcano plot. **d** GO analysis of downregulated genes in Malat1^−/−^ MOVAS cells compared with the WT type. **e** Qualitative PCR analysis of RNA levels of *MMP2*, *MMP9*, *IL-1β*, and *TNF-α* in KO and WT MOVAS cells at baseline and with AngII stimulation. *n* = 5 per group. Corresponding proteins were detected by immunoblotting. *n* = 5 per group. **f** The relative RNA levels of *Malat1*, MMPs, and inflammatory factors were detected in DMSO and MALAT1-IN-1 groups administered Ang II. *n* = 5 per group. Corresponding proteins were detected by immunoblotting. *n* = 5 per group. Data were presented as mean ± SEM and normal distributions were tested by the Shapiro–Wilk method, which showed that all data were normally distributed. Two-way ANOVA followed by Sidak post hoc test used for (**e**, **f**). *P < 0.05, **P < 0.01, ***P < 0.001, ****P < 0.0001. Each experiment was repeated independently for a minimum of three times

The key mechanisms widely accepted for AAA include inflammatory infiltration and matrix degradation mediated by MMPs.^7,36^ To explore the role and pathogenesis of *Malat1* in AngII-induced AAA formation, we constructed a mouse aortic vascular smooth muscle cell line with stable knockout of the *Malat1* gene. *Malat1* gene-related sequencing and northern blotting confirmed the successful knockout in this cell line (Malat1^−/−^ smooth muscle cells; Fig. 6a).

Malat1^−/−^ and WT (control) smooth muscle cells at baseline and with AngII stimulation were sequenced by RNA transcriptome sequencing to detect the corresponding gene changes (Fig. 6b). The downregulated genes occupied a dominant position with a total of 2396 after *Malat1* knockout, in which the expression of MMPs, chemokines and inflammatory factors was significantly reduced (Fig. 6b, c). In addition, AngII stimulus aggravated the expression levels of these genes. In contrast, genes associated with the regulation of blood pressure and negative regulation of inflammation, such as the genes *Calca* and *Tmc7,*^37^ were upregulated (Fig. 6c).

According to the pathway analysis of downregulated genes, the KO cells were enriched in distinct pathways involved in TNF and AMPK signaling^38^ (Supplementary Fig. S7a). Simultaneously, the biological processes for negative regulation of smooth muscle cell contraction and metal ion binding played a key role in VSMC functional disorders and extracellular matrix degradation (Fig. 6d). Therefore, the AngII-induced pathways were attenuated by *Malat1* knockout.

Thereafter, we verified the expression level of MMPs and inflammatory factors when *Malat1* was inhibited in vitro. The knockout of *Malat1* suppressed MMPs (MMP2, MMP9) and inflammatory factor (IL-1β, TNF-α) expression at both the mRNA and protein levels (Fig. 6e). A new inhibitor of *Malat1* (MALAT1-IN-1)^39^ was used in the VSMCs with a concentration of 10^−5^ M/ml, and the RNA level of *Malat1* was reduced by nearly 50%; moreover, the mRNA and protein levels of MMP2, MMP9, IL-1β, and TNF-α were also significantly reduced (Fig. 6f). In summary, *Malat1* promoted the expression of MMPs and inflammatory infiltration in VSMCs.

### The inhibitor of *Malat1* can significantly prevent, inhibit, and reverse AngII-induced AAA in vivo

**Fig. 7:**
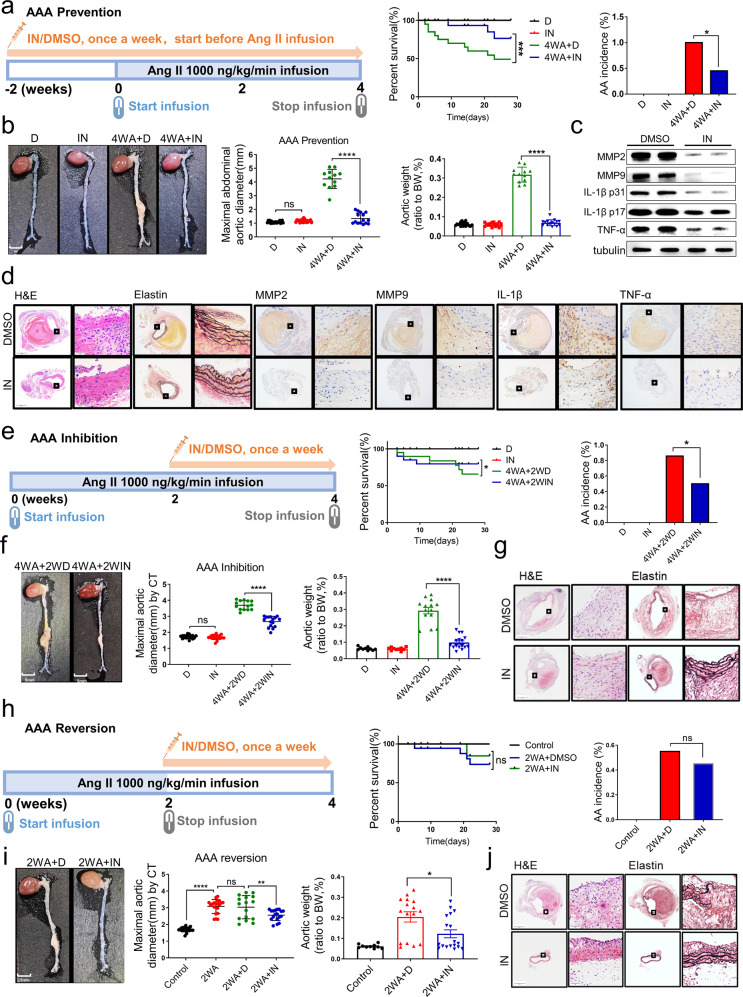
An inhibitor of *Malat1* prevented, inhibited, and reversed AngII-induced AAA in vivo. **a** Diagram of the experimental design for the preventive effect of the inhibitor (MALAT1-IN-1). The preventive effect of the inhibitor on AAA formation was significant with injection 2 weeks prior to AngII infusion. The results of the survival curve and the AAA incidence supported the preventive effect of the inhibitor. *n* = 20 per group. **b** The preventive effect of the inhibitor on AAA formation was revealed based on macroscopic histopathology in vivo. The maximal abdominal aortic diameter and the total aortic weights in mice injected with inhibitor were lower than those in the DMSO group. *n* = 20 in both D group and IN group. *n* = 12 in 4WA + D group and *n* = 15 in 4WA + IN group. **c** MMP2, MMP9, IL-1β, TNF-α, and tubulin protein expression in the abdominal aortas of indicated groups. *n* = 5 per group. **d** Representative staining with H&E, elastin in the abdominal aortas of indicated groups. The magnification of the two insets was 40-fold and 400-fold. **e** Diagram of the experimental design for the inhibitory effect of the inhibitor. The inhibitory effect of the inhibitor administered over the last 2 weeks of the 4-week continuous AngII infusion. The results of the survival curve and AAA incidence supported the inhibitory effect of the inhibitor. *n* = 20 per group. **f** The inhibitory effect of the inhibitor on AAA progression was revealed based on macroscopic histopathology in vivo. The maximal abdominal aortic diameter and the total aortic weights in mice injected with the inhibitor were lower than those in the DMSO group. *n* = 20 in both D group and IN group. *n* = 14 in 4WA + 2WD group and *n* = 16 in 4WA + 2WIN group. **g** Representative H&E and elastin staining in the abdominal aortas of indicated groups. **h** Diagram of the experimental design for the reversal effect of the inhibitor. The reversal effect of the inhibitor on AAA was tested by injecting it over the last 2 weeks without AngII infusion. The results of the survival curve and the AAA incidence supported the reversal effect of the inhibitor. *n* = 20 per group. **i** The reversal effect of the inhibitor on AAA was shown based on macroscopic histopathology in vivo. The maximal abdominal aortic diameter and the total aortic weights in mice injected with the inhibitor were lower than those in the DMSO group. *n* = 20 in both the control group and 2WA group. *n* = 16 in 2WA + D group and *n* = 18 in 2WA + IN group. **j** Representative H&E and elastin staining in the abdominal aortas of indicated groups. The control groups without angiotensin II were included with all results to compare the effect of the inhibitor. D, DMSO; IN, inhibitor; 4WA + D, 4-week Ang II + DMSO; 4WA + IN, 4-week Ang II + inhibitor. 4WA + 2WD, 4-week Ang II + 2-week DMSO; 4WA + 2WIN, 4-week Ang II + 2-week inhibitor. 2WA + D, 2-week Ang II + DMSO; 2WA + IN, 2-week Ang II + inhibitor. (*n* = 20 for each group). Data were presented as mean ± SEM and normal distributions were tested by the Shapiro–Wilk method, which showed that all data were normally distributed, except (**i**) (right). Log-rank test was used for the survival curve in (**a**, **e**, **h**). Differences in the incidence were analyzed with the chi-squared test in (**a**, **e**, **h**). One-way ANOVA followed by Tukey post hoc test was used for (**b**, **f**, **i**) (middle). Kruskal–Wallis test was used for (**i**) (right). *P < 0.05, **P < 0.01, ***P < 0.001, ****P < 0.0001. Each experiment was repeated independently for a minimum of three times

To explore the preventive effect of the *Malat1* inhibitor in vivo, we constructed an AAA model via AngII subcutaneous pump infusion in ApoE^−/−^ male mice fed a high-fat diet. The mice were divided into four groups, DMSO (D), inhibitor (IN), 4-week Ang II + DMSO (4WA + D) and 4-week Ang-II + inhibitor (4WA + IN) groups. The inhibitor was injected for 2 weeks before AngII infusion and continued for the whole process. At the end of the experiment, the number of surviving mice in the 4WA + IN group was higher than that in the 4WA + D group (Fig. 7a). The incidence of AAA and other basic indicators of AAA evaluation were significantly improved by the inhibitor (Fig. 7b). Previous studies found that degradation of the extracellular matrix in AAA was attributed to upregulated MMPs, particularly MMP2 and MMP9.^40^ Macrophage infiltration and chronic inflammation of the aortic wall are other pathological features of AAA.^41^ Therefore, we examined the protein expression of MMPs and inflammatory cytokines by western blotting and immunohistochemistry (Fig. 7c, d), which were markedly and significantly reduced by the inhibitor (*P* < 0.01, one-way ANOVA; Supplementary Fig. S7b, c). HE and elastin staining showed that these pathological changes occurred more frequently with a higher grade of elastin degradation in the 4WA + D group (Supplementary Fig. S7d). These results suggest that MALAT1-IN-1 effectively prevented the pathological remodeling of the aortic wall induced by AngII infusion.

To explore the inhibitory and reversing effect of the inhibitor, we further designed inhibitory and reversal animal experiments respectively to evaluate the inhibitor in AAA protection. In the inhibitory experiment, the mice were randomly divided into two groups (*n* = 20) and infused with Ang II for 4 weeks. During AngII infusion, the two groups were separately injected with inhibitor or DMSO for the last 2 weeks (Fig. 7e). In the reversal experiment, the mice were randomly divided into two groups (*n* = 20) and infused with AngII for 2 weeks. Then the osmotic pump was removed to stop AngII infusion, and mice were separately injected with inhibitor or DMSO for another 2 weeks (Fig. 7h).

Statistical analysis of changes in the diameter of the mice in the same group based on CT imaging, showed that the inhibitor could reverse the established AAA or the widen aorta (Supplementary Fig. S7e, f). The AAA evaluation indicators (survival curves, AAA incidence, aortic diameter, etc.) and histopathology results demonstrated that the inhibitor could effectively reverse and inhibit AngII-induced AAA (Fig. 7e–j and Supplementary Fig. S7g, h). Ultimately, these results clarified the preventive, inhibitory and reversing effect of the inhibitor and provided new evidence for its treatment value in further clinical applications.

### Single-cell analysis of AngII-induced AAA with DMSO or inhibitor revealed the cell composition and gene expression changes

First, VSMC 4 with high *Malat1* expression were mainly distributed in the abdominal aorta in the healthy mice, which provided the basic condition for the occurrence of AngII-induced AAA. To analyze the cell composition and gene expression changes at the single-cell level, we used single-cell sequencing to detect the AAA tissues with or without the inhibitor and verified the aforementioned conclusions.

**Fig. 8:**
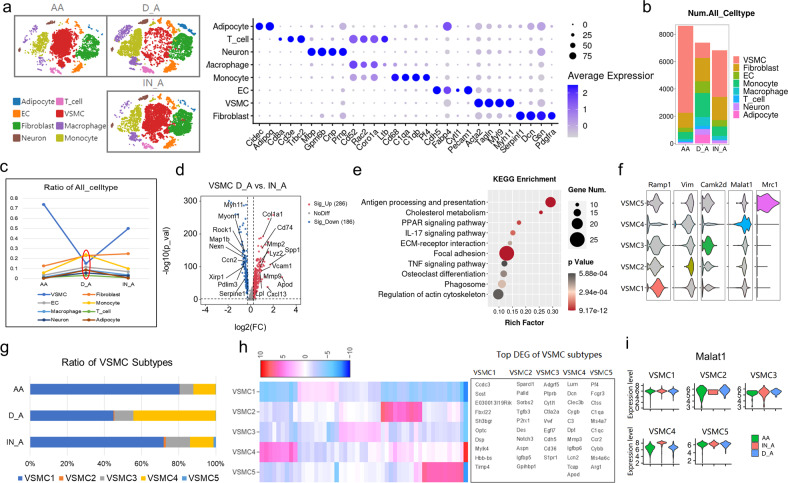
Single-cell analysis of AngII-induced AAA with DMSO or inhibitor. **a** T-SNE plot showing the clustering of 22, 975 cells isolated from aortic tissues of three groups by split view. The dot plot showed that the average scaled expression levels of marker genes were consistent with the identification of the first five samples. AA, healthy abdominal aorta; D_A, AngII-induced AAA with DMSO; IN_A, AngII-induced AAA with inhibitor. **b** The bar chart showed the number of major cell types across the three groups. **c** The line chart showed the proportion of major cell types across the three groups. The ratio of VSMCs and immune cells changed the most. **d** The fold change of all genes with a significant difference in VSMCs between D_A and IN_A groups was presented by the volcano plot. **e** KEGG analysis of downregulated genes in IN_A vs. D_A group. **f** Identification of VSMC subtypes based on the previous marker genes in the first five samples. **g** The bar chart showed the ratio of five VSMC subtypes across three groups, in which VSMC 1 and VSMC 4 changed obviously. **h** In addition to the existing marker genes, the top differentially expressed genes (DEGs) in each subtype were shown by the heatmap. **i** The expression level changes of *Malat1* in each subtype across three groups are shown by the violin plot, in which the *Malat1* expression level of VSMC 4 increased in the D_A group

According to the new scRNA-seq data after batch correction, we obtained 22, 975 cells totally isolated from aortic tissues of three groups including the healthy abdominal aorta group (AA group), Ang II + DMSO group (D_A group), and AngII + Inhibitor group (IN_A group) (Fig. 8a). The results identified a total of eight cell types, including fibroblasts (*Dcn*, *Pdgfra*), VSMCs (*Tagln*, *Myl9*), ECs (*Cdh5*, *Pecam1*), and monocytes (*C1qa*, *Pf4*), among others. The dot plot showed that the average scaled expression level of marker genes was consistent with the identification of the first five samples (Fig. 8a). Samples from the three different groups shared similar cell patterns but had different cell frequencies. The number and ratio of VSMCs obviously decreased in the abdominal aortic aneurysm (D_A group) compared with those in the AA group but were recovered by the inhibitor (Fig. 8b, c). However, immune cells such as monocytes exhibited the exact opposite changes (Fig. 8b, c).

In the volcano plot, the expression of genes associated with the SMC contractile phenotype (*Rock1*, *Myh11*) was downregulated, whereas that of the AAA-related genes (*Mmp2*, *Spp1*) was upregulated in the D_A group compared with the IN_A group (Fig. 8d). Correspondingly, the KEGG analysis of downregulated genes in the IN_A group compared with the D_A group indicated that they were enriched in pathways including ECM–receptor interaction and TNF signaling pathway (Fig. 8e). These results indicated that the inhibitor could alleviate extracellular matrix degradation and the reduction of the SMC contractile phenotype.

To clarify whether the VSMC 4 with high *Malat1* expression contributed to aortic dilation, we defined the VSMC subtypes with the same markers used in the first five samples and successfully reproduced the five main subtypes (Fig. 8f). The percentage of subtypes changed significantly with different trends. VSMC 1 showed the most obvious reduction in the D_A group, which was rescued by the inhibitor (Fig. 8g). In contrast, VSMC 4 were predominant in terms of their ratio in the D_A group and returned to normal levels in the IN_A group (Fig. 8g). The top expressed genes of the heatmap also showed that VSMC 4 highly expressed the AAA-related gene *Mmp3* (Fig. 8h). Further, the expression level of *Malat1* was significantly higher in VSMC 4 compared with the AA group, whereas the other subtypes performed with no significant differences (Fig. 8i). In conclusion, VSMC 4 highly expressing *Malat1* predominantly contributed to AngII-induced AAA and the inhibitor could rescue the reduction of VSMCs to alleviate the AAA lesion.

### Cell-to-cell interaction analysis with WGCAN and Circos plots

WGCAN analysis revealed all gene modules expressed among all cell types. This provided further evidence that even different cell types, including structural cells and immune cells, could demonstrate similar gene expression patterns, such as VSMC 5, monocytes, and T cells (Supplementary Fig. S8a). However, different subclusters of the same cell type shared the same or overlapping gene patterns, such as VSMCs 2, 3, and 4. These features were also supported by sample clustering analysis (Supplementary Fig. S8a).

In the cell-to-cell interaction network, a closer position to the center with lines connected to cell types indicated a more important status occupied (Supplementary Fig. S8b). EC1 and VSMC 4 were situated in the middle of all cell types, which indicates their essential effect on the physiological and pathological processes in the aorta (Supplementary Fig. S8c). In addition, neurons with upstream signal regulation using electrical signals and transmitters, should not be ignored. It was observed that VSMC 3 was located between VSMCs 2 and 4, with the role of connection and transition as described previously (Supplementary Fig. S8c).

Thereafter, we analyzed the specific gene interactions among all cell types and selected all the VSMCs separately with Circos plots (Supplementary Fig. S8d). The different genes and genes expressed between cell subtypes also had close or isolated relationships across cell type boundaries. Therefore, subtypes of the same cell type were expressed differently, whereas the subtypes of different cell types had very similar expression patterns and functions. All these analyses of cell-to-cell interactions provided further evidence of the heterogeneity of the aorta, whether based on subtypes or spatial distribution.

## Discussion

Regardless of the blood vessels of mice or humans, the research basis of various vascular diseases is determining the characteristics of blood vessel composition at the physiological level. The anatomical structure and origin of embryonic development create heterogeneity in the aortic microenvironment. This study provided gene expression and cell subtype information for ~26,000 cells in total and ~5000 cells in each segment on average based on the spatial dimension. The EC subtypes identified in our study were matched with those from a previous study.^11^ In addition to the discovered effects of EC subtypes, the new segmental characteristics of EC subtypes were revealed; in particular, EC1 highly expressed *Vcam1* and *Scarb2* in the aortic arch. In fibroblasts, Fbn1^+^ Fibro1 was identified and showed high expression levels in the thoracic aorta, particularly in the AOAR segment. This result provided a possible and reasonable explanation that the specific distribution of Fbn^+^ Fibro1 might be an important physiological basis for the position of thoracic aortic aneurysm (TAA) occurrence. It also emphasized disease susceptibility and possible dominant cell subtypes, which required further research and discussion in the future.

This study focused on the three VSMC subtypes with significant differences in spatial distribution. The location of VSMC 4 gathering was the abdominal portion, which was shown to have a high incidence of aortic aneurysms in the AngII infusion model. Therefore, the top marker gene *Malat1* of VSMC 4 was detected by FISH at both transverse sections and tissue fragment levels, and this resulted in significant distribution differences in VSMCs. The RNA-sequencing database of human aortic tissues and previous studies also provided evidence of *Malat1* upregulation in the AAA group. We explored the role of *Malat1* in vascular smooth muscle cells both in vitro and in vivo. Although, it was more insightful by using SMC fate mapping mice which was published by Dobnikar et al. in 2018.^32^ The advantages of SMC fate mapping mice were obvious in separation and positioning of smooth muscle cells both in vivo and in vitro. We also used a variety of experimental methods to verify and explore the mechanism underlying the key VSMC subtype, including FISH, single-cell sequencing, and a series of animal experiments, which were investigated from different perspectives.

For mechanistic research on *Malat1*, the previous study resulted in the important conclusion that a HDAC9-MALAT1-BRG1 complex regulated thoracic aneurysm progression^19^ which provided the foundation for our present study. However, there were many differences in the role of MALAT1 in AAA formation and VSMC dysfunction in our study compared with that in TAA. In animal model construction, the TAA model is a kind of spontaneous lesion of the thoracic aorta in Marfan (Fbn1^C1039G/+^) mice. The mechanism is that Fibrillin gene mutations cause enhanced TGF-β signaling. In contrast, the AAA model chosen in this study was an AngII-induced AAA model with high-fat feeding, as the most typical and generally accepted animal model of AAA, which mimics most human AAA features. In addition, another key mechanism underlying AAA is increased inflammatory factor infiltration including TNF-α and IL-1β, as compared to that with TAA. Chronic inflammation of the aortic wall in AAA was determined to be related to *Malat1* in our study, which was quite different from TAA.

Moreover, we demonstrated for the first time that a *Malat1* inhibitor can effectively protect the aorta based on three aspects, namely the prevention, inhibition, and reversal of AAA. The novel application of the *Malat1* inhibitor had significant clinical value in therapeutic strategies for AAA. Our data demonstrated that the physiological high level of *Malat1* in the abdominal aorta was an important reason underlying the susceptibility to AngII-induced AAA. Further, we used single-cell analysis to confirm the dominant status of VSMC 4 with high *Malat1* expression in AAA and the therapeutic effect of the inhibitor at the single-cell level.

In conclusion, our study provides a comprehensive segmental single-cell transcriptome of the aorta at the physiological level, inspiring new ideas for diagnostic and intervention targets for aortic diseases. Furthermore, a novel system of VSMCs was proposed to replace the traditional classification. The pivotal subtype VSMC 4, with the top marker gene *Malat1*, can be a new target for AAA with the potential therapeutic inhibitor, MALAT1-IN-1 already available.

## Materials and methods

The “Materials and methods” section is available in Supplemental Materials.

## Supplementary information

Supplementary Materials
