# Supplementary material for: An intersegmental single-cell profile reveals aortic heterogeneity and identifies a novel Malat1+ vascular smooth muscle subtype involved in abdominal aortic aneurysm formation
Source: Signal Transduct Target Ther. 2022 Apr 27;7:125. doi: 10.1038/s41392-022-00943-x (PMC9043217; doi:10.1038/s41392-022-00943-x)
Supplement: Supplementary file 1 — Supplementary Materials [file 41392_2022_943_MOESM1_ESM.docx]

Supplementary Materials for

An intersegmental single-cell profile reveals aortic heterogeneity and identifies a novel Malat1^+^ vascular smooth muscle subtype involved in abdominal aortic aneurysm formation

Liwen Yu^1^, Jie Zhang^1^, Amy Gao^1^, Meng Zhang^1^, Zunzhe Wang^1^, Fangpu Yu^1^, Xiaobin Guo^2^, Guohai Su^2^, Yun Zhang^1,2^, Meng Zhang^1,2*^ and Cheng Zhang^1,2*^

Correspondence to: [zhangmeng@sdu.edu.cn](mailto:zhangmeng@sdu.edu.cn), [zhangc@sdu.edu.cn](mailto:zhangc@sdu.edu.cn)

**This PDF file includes:**

Materials and Methods

Supplementary Text

Figures. S1 to S8

Tables S1 to S4

Materials and Methods

**Animal**

All animal care and experimental protocols were approved by the Ethics Committee on Animal Experiment of Shandong University Qilu Hospital and complied with the guidelines of the Animal Management Rules of the Chinese Ministry of Health (Document No. 55, 2001). Animal studies were reported in compliance with the ARRIVE guidelines (Kilkenny, Browne, Cuthill, Emerson, & Altman, 2010) and with the recommendations made by the British Journal of Pharmacology. All of the C57/BL6 and ApoE^-/-^ mice aged 8 weeks were purchased from Beijing Weishanglide Animal Experimental Center. All mice were housed in standard cages in a specific pathogen-free environment and kept on a 12-hr light/12-hr dark cycle with food and water freely available. The whole aortas isolated form five male C57/BL6 mice were divided into five segments by anatomical landmarks and digested to obtain a single cell suspension.

In the *in vivo* study, the ApoE^-/-^ mice were randomly divided into two groups, Ang II group and control group (n = 30 per group). All mice were treated for 4 weeks with continuous subcutaneous infusion by an osmotic pump (Alzet model 2004, Alza Corp, Palo Alto, CA, USA) with high-fat feeding at the same time. The control group received an infusion of saline while the Ang II group received an infusion of Ang II (1,000 ng/kg/min^1^, MCE, Cat. No.: HY-13948) for 4 weeks. In the mechanism experiment, the mice were divided into four groups, DMSO group (D), inhibitor group (IN), 4-week Ang II+ DMSO group (4WA+D) and 4-week Ang II+ inhibitor group (4WA+IN). The inhibitor was injected for 2 weeks before the Ang II infusion and continued for the whole process (n = 20 per group). The mice were separately injected via tail vein twice a week with MALAT1-IN-1 solution (200ug/kg, MCE, Cat. No. HY-115579) and DMSO diluted by cosolvent in same concentration of 2%. In the inhibitory experiment, the ApoE^-/-^ mice were randomly divided into 2 groups (n = 20) and were infused with Ang II (1,000 ng/kg/min) by osmotic pumps with high-fat feeding at the same time for 4 weeks. During the infusion of Ang II, the two groups were separately injected with inhibitor or DMSO for the last 2 weeks (Fig. 7e). In the reversal experiment, the ApoE^-/-^ mice were randomly divided into 2 groups (n = 20) and were infused with Ang II (1,000 ng/kg/min) by osmotic pumps with high-fat feeding at the same time. After 2 weeks, the osmotic pump was taken out and the Ang II infusion was stopped. Then, the two groups were separately injected with inhibitor or DMSO for another 2 weeks. All mice were humanely killed (overdose of pentobarbital) and samples taken for further histological studies.

MALAT1-IN-1 (compound 5) is a potent and specific Malat1 (Metastasis-associated lung adenocarcinoma transcript 1) inhibitor. MALAT1-IN-1 modulated Malat1 downstream genes in a dose-dependent manner without affecting expression of nuclear enriched abundant transcript 1 (Neat1)^2^. The specificity of the inhibitor for Malat1 was demonstrated by nuclear magnetic resonance spectroscopy. Small molecules specifically targeting the Malat1 ENE triplex lay the foundation for new classes of anticancer therapeutics and molecular probes for the treatment and investigation^2^. The dose of the inhibitor was referred to that used in MMTV-PyMT tumors with a final concentration of 1 μM. The blood volume was calculated (1.5ml-2ml) according to mouse body weight (20-25g) and the injection dose was increased appropriately on the basis.

**Tissue isolation and cell dissociation.**

The whole aorta was dissected from the aortic root (exclude aortic valves) to arteria iliaca communis. The isolated aorta included five portions, aortic arch, thoracic aorta 1, 2, and abdominal aorta 1, 2 segments. Perivascular adipose and connective tissues were separated and removed from the vascular tissue before dissociation and single-cell analysis. The single cell suspension of each portion was obtained by enzymatic digestion referred to the protocol^3^ previously described. The enzyme digestion program was improved and adjusted in detail as followed: the aortic tissues were cut into 0.5 mm2 pieces and digested in enzyme solution (1mg/ml collagenase I, 0.5 mg/ml Elastase, 1mg/ml DNase I) in 37 ℃ water bath with shaking for 40 min at 100 rpm. Digestion was terminated with 1× PBS containing 10% fetal bovine serum (FBS) and pipetted for 5-10 times. The cell suspension was filtered by passing through 30um stacked cell strainer and centrifuged at 300g for 5 min at 4 °C. The cell pellet was resuspended in 100ul 1× PBS (0.04% BSA). The overall cell viability was more than 90% confirmed by trypan blue exclusion. The single cell suspensions were counted using Countess II Automated Cell Counter and the concentration was adjusted to 700-1200 cells/μl before single cell analysis.

**Single-cell RNA-seq library preparation and sequencing**

Single-cell suspensions were loaded to 10x Chromium to capture 5000 single cells according to the manufacturer’s instructions of 10X Genomics Chromium Single-Cell 3’ kit (V3). The following cDNA amplification and library construction steps were performed according to the standard protocol. Libraries were sequenced on an Illumina NovaSeq 6000 sequencing system (paired-end multiplexing run,150bp) by LC-Bio Technology Co., Ltd (Hang Zhou, China). For quality control, cells with >25% reads mapping to mitochondria, or with <700 or >15,000 UMI counts indicated low quality, and were removed from the analysis. Then the Doublet Finder R package was used to filter for the second time to remove the doublets.

CellRanger software (10x Genomics) was then used to analyze the sequencing data and produced gene expression information for each cell. Cell Ranger (http://support.10xgenomics.com/single-cell/software/overview/welcome) used an aligner called STAR (https://github.com/alexdobin/STAR), which performed splicing-aware alignment of reads to the genome. Cell Ranger then used the transcript annotation GTF to bucket the reads into exonic, intronic, and intergenic, and by whether the reads align (confidently) to the genome. A read was exonic if at least 50% of it intersects an exon, intronic if it is non-exonic and intersects an intron, and intergenic otherwise.

To overcome the extensive technical noise in any single gene for scRNA-seq data, Seurat clusters cells based on their PCA scores, with each PC essentially representing a ‘metagene’ that combines information across a correlated gene set. Determining how many PCs to include downstream is therefore an important step. In Macosko et al, we implemented a resampling test inspired by the jackStraw procedure. We randomly permute a subset of the data (1% by default) and rerun PCA, constructing a ‘null distribution’ of gene scores, and repeat this procedure. We identify ‘significant’ PCs as those who have a strong enrichment of low p-value genes.

**Differentially expressed genes GO and KEGG enrichment analysis**

GO enrichment analysis provides all GO terms that significantly enriched in differentially expressed genes comparing to the genome background, and filter the differentially expressed genes that correspond to biological functions. Firstly, all peak related genes were mapped to GO terms in the Gene Ontology database (http://www.geneontology.org/), gene numbers were calculated for every term, significantly enriched GO terms in differentially expressed genes comparing to the genome background were defined by hypergeometric test.

Genes usually interact with each other to play roles in certain biological functions. Pathway- based analysis helps to further understand genes biological functions. KEGG is the major public pathway-related database. Pathway enrichment analysis identified significantly enriched metabolic pathways or signal transduction pathways in differentially expressed genes comparing with the whole genome background.

The personalized analysis including WGCAN, branching gene expression trajectory analysis and Cell-to-cell interaction analysis. Weighted gene co‐expression network analysis (WGCNA) was applied to the count per million (CPM) expression data. A co‐expression network was constructed with a beta value of 20. Genes were clustered into branches of highly expressed genes, and modules were identified with the tree cut algorithm with the additional PAM stage. Branching gene expression trajectory analysis was performed with using the Monocle2 R package (Trapnell et al., 2014). Top 50 highly variable genes from the “FindMarkers” function of Seurat package were used as ordering genes with the threshold of adjusted P value < 0.05 and |logFC| > 1. Cell-to-cell interaction analysis was performed using the CellPhoneDB (version 1.1.0). Only receptors and ligands expressed in more than 10% cells of any cell type were further evaluated. Only those with a P value < 0.01 were used for the prediction of cell-cell interaction between any two cell types.

**Immunofluorescence localization, Fluorescence in situ hybridization (FISH) and Whole-Mount Fluorescence in situ hybridization (WISH)**

Immunofluorescence localization was performed by TSA Plus double immunofluorescence labeling staining kit (Servicebio, G1226-50T) using primary antibodies including Cd31 antibodies (Abcam, ab182981), Vcam1 antibodies (CST, 39036), Scarb2 antibodies (CST, 27960), Pdgfra antibodies (CST, 3174), Fbn1 antibodies (Bioss, bs-1157R), Sm22-α antibodies (Abcam, ab14106). The secondary antibodies contained HRP-conjugated Affinipure Goat Anti-Rabbit IgG(H+L) (Proteintech, SA00001-2), Donkey Anti-Rabbit IgG H&L (Alexa Fluor® 594, ab150068), Donkey Anti-Rabbit IgG H&L (Alexa Fluor® 488, ab150065), and Donkey Anti-Mouse IgG H&L (Alexa Fluor® 488, ab150109). In situ hybridization was performed as described previously. In order to study the cell type distribution of Malat1, RNAScope in situ hybridization was performed^4^ per the manufacturer's instructions using a purposed probe (Malat1, Advanced Cell Diagnostics, catalog no. 313391) and the RNAScope 2.5 HD reagent kit-RED (ACD, cat.no. 322350). Probes targeting the bacterial gene dapB (ACD, catalog no. 310043) were used as negative control. Probe targeting Malat1 was detected in lung tissue as positive control. Slides were mounted with VectaShield mounting medium containing DAPI (Vector Laboratories) and then imaged under both fluorescence and brightfield conditions. The Whole-Mount Fluorescence in situ hybridization was performed referred to the previous study of vessels in embryo^4^ and replaced by using the same probes and regent kit in FISH.

**Cell culture and experiment in vitro**

In vitro study, mouse aortic vascular smooth muscle cells, MOVAS were purchased from American Type Culture Collection (Manassas, VA, USA) and cultured in high-glucose DMEM supplemented with 10% fetal bovine serum and 1% penicillin/streptomycin. The MOVAS cells were divided into control group and Ang II-stimulated group (10^-6^ M/ml) and incubated for 24 or 48 hours.

The MOVAS cells with stable knockout of Malat1 gene using CRISPR/Cas9 system by lentiviral transfection. Three pairs of small-guide RNA (sgRNA) targeting the Malat1 gene were designed and inserted into plasmid pLenticrisprV2. The sg2 and sg6 were selected with the best performance as expected (Table S1). The recombinant plasmid pLenticrisprV2 containing sgRNA was packaged using a lentivirus packaging system. MOVAS cells were transfected with viruses, and monoclonal cells were screened using puromycin. The genomic DNA was extracted from the amplified monoclonal cells. Eventually the Malat1 gene-related sequencing and the electrophoresis band of Northern-blot compared with the wild-type Malat1 gene confirmed the cell line with successful knockout (Malat1^-/-^ MOVAS cells)

**Bulk gene expression analysis**

Total RNA of KO and WT MOVAS cells was isolated and purified using TRIzol reagent (Invitrogen, Carlsbad, CA, USA) following the manufacturer's procedure. The RNA amount and purity of each sample was quantified using NanoDrop ND-1000 (NanoDrop, Wilmington, DE, USA). The ligated products are amplified with PCR by the following conditions: initial denaturation at 95℃ for 3 min; 8 cycles of denaturation at 98℃ for 15 sec, annealing at 60℃ for 15 sec, and extension at 72℃ for 30 sec; and then final extension at 72℃ for 5 min. The average insert size for the final cDNA library was 300±50 bp. At last, we performed the 2×150bp paired-end sequencing (PE150) on an Illumina Novaseq™ 6000 following the vendor's recommended protocol.

**Quantitative real-time PCR**

The extracted RNA was dissolved in RNase free water, and concentration of the total RNA was determined with a spectrophotometer. Total RNA (1ug) was reverse transcribed into cDNAs with the PrimeScript™ RT Reagent Kit (Takara, Japan). qRT-PCR was performed using SYBR Green (Takara, Japan), and the results were normalized to β-actin expression. All primer sequences are shown in Supplemental table 1.

**Western blotting**

Proteins were extracted from cells or fresh tissue lysates of the abdominal aortas. The corresponding primary antibodies were tubulin antibody (Proteintech, 10094-1-AP), MMP2 antibody (CST,40994), MMP9 antibody (R&D, AF909-SP), IL-1β antibody (Abcam, ab9722), TNF-α antibody (Abcam, ab6671). The levels of protein expression of MMP2, MMP9, IL1β and TNF-α were normalized to that of tubulin. Three independent experiments were performed to derive the mean values calculated for statistical analysis.

**Histology and immunohistochemical staining**

At the end of the study, the mice were killed by an overdose of pentobarbital (i.p.). The abdominal aortas were removed and fixed in 4% paraformaldehyde overnight. Then, the aortic tissues were embedded with paraffin and cut into 5-μm-thick cross sections, which were stained with HE and Weigert stains in accordance with standard protocol^5^. To quantitate elastin degradation, a previously described grading method was used, Grade 1: no elastin degradation; Grade 2: mild elastin degradation; Grade 3: severe elastin degradation; and Grade 4: aortic rupture.

For immunohistochemical staining, the corresponding primary antibodies were MMP2 antibody (CST,40994), MMP9 antibody (R&D, AF909-SP), IL-1β antibody (Abcam, ab9722), TNF-α antibody (Abcam, ab6671). The positive reactions of tissue sections were developed using the peroxidase substrate solution including 0.02% H2O2 and 0.1% 3,30 -diaminobenzidine tetrahydrochloride (ZLI-9018, ZSGB-BIO, China) with the reaction products displayed as a brown color. Finally, the sections were counterstained with haematoxylin, and the integration optical density values of positive staining were calculated using Image-Pro Plus software (Media Cybernetics).

**In Vivo Computed Tomography (CT) Imaging**

To evaluate the aortic diameter and pathology in vivo, the high-resolution contrast-enhanced CT imaging was performed in mice of the four groups at 0 week, 2week and 4weeks separately before each operation. After mice were anesthetized with isoflurane, imaging was performed with Quantum GX2 Micro-CT scanner (PerkinElmer, Inc., Waltham, MA). Mice were intravenously administered a liposomal-iodine (liposomal-I, 1.1 g I/kg) contrast agent for CT angiography. The X-ray system of the scanner used a microfocus tube with a focal spot size of 5 um at 4 W with 7 minutes scan time. CT images were analyzed in Materialise Mimics (version 20.0 X64) to measure the diameter of the aorta for the presence of dilation, aneurysm, or dissection.

**Statistical Analysis**

Marker genes for transcriptional subpopulations in scRNA-seq profiles were identified with the FindAllMarkers Seurat function with a minimum log-fold change threshold of 0.25 and with P values computed with a Wilcoxon rank-sum test. P values were computed with a hypergeometric test and adjusted for multiple hypothesis correction with a Benjamini-Hochberg procedure. Gene set scores and imaging characteristics were compared between cell populations with the Mann–Whitney U test. All data were presented as mean ± SEM. Shapiro-Wilk test was first used to assess Gaussian distribution. All analyses were performed with GraphPad Prism 8 (GraphPad, San Diego, CA). Each experiment was repeated independently for a minimum three times. And For data with Gaussian Distribution, one-way ANOVA followed by Tukey post hoc tests were performed to determine the statistical difference between multiple groups with one variable and normally distribution. To compare multiple groups with more than one variable, two-way ANOVA followed by Sidak post hoc test was used. For data with a non-Gaussian distribution, Kruskal-Wallis test was used for multiple group comparisons. Differences in the incidence and grading of AAA were analyzed with chi-squared test. In all statistical comparisons, a p value of < 0.05 was considered statistically significant. Unless otherwise specified, each legend displays an adjusted P value for multiple comparisons. Non-significant p-values were not shown.

**Data Availability**

The scRNA-seq data generated in this study have been deposited in the Gene Expression Omnibus (GEO) database under the accession code GSE193265 and GSE191226. All other data supporting the findings of this study are available from the corresponding author on reasonable request.

**References**

1 Wang, S. *et al.* Activation of AMP-activated protein kinase α2 by nicotine instigates formation of abdominal aortic aneurysms in mice in vivo. *Nat Med*. **18**, 902-910, (2012).

2 Butcher, M. J., Herre, M., Ley, K. & Galkina, E. Flow cytometry analysis of immune cells within murine aortas. *J Vis Exp*, (2011).

3 Herriges, M. J. *et al.* Long noncoding RNAs are spatially correlated with transcription factors and regulate lung development. *Genes Dev*. **28**, 1363-1379, (2014).

4 Ramirez, A. & Astrof, S. Visualization and Analysis of Pharyngeal Arch Arteries using Whole-mount Immunohistochemistry and 3D Reconstruction. *J Vis Exp*, (2020).

5 Satoh, K. *et al.* Cyclophilin A enhances vascular oxidative stress and the development of angiotensin II-induced aortic aneurysms. *Nat Med*. **15**, 649-656, (2009).

Supplementary Text

**Fig. S1. Quality Control (QC) plots.**

**a** Fraction Reads in Cells of whole samples were ideally higher than 70%. The barcodes corresponding to the blue line were effective cells, and the gray line was background noise. **b** The number above the figure was the Pearson correlation coefficient between the number of UMI and genes in five samples. The closer the correlation coefficient was to 1, the stronger the correlation was. **c** Violin plots showing the distribution of the number of genes, number of counts, and percentage of mitochondrial fraction in all five samples. To reduce the number of low-quality cells, doublet and multi-cell ratio, cells were filtered out if more than 2,0000 counts, less than 500 genes were detected, and the percentage of mitochondrial gene counts higher than 25%.

**Fig. S2. Ang II induced AAA mice model and cell composition of all aortic segments**

**a** The gross pathology and incidence of aortic aneurysm in different segments. **b** All clusters were obtained by single-cell analysis in a t-SNE plot. The 15 cell types were identified with classic marker genes by CellRanger software. **c** The main cell types are distributed evenly in general across five segments. n = 30 per group. **d** The cell number of all subtypes in five segments. The asterisks in **Fig. S2d**, symbols to highlight the subtypes with number differences across five segments. ****P<0.0001 by chi-squared test in **Fig. S2a**.

**Fig. S3. The marker genes identified ECs subtypes and related KEGG analysis**

**a** The t-SNE plot showed the expression level of marker genes in EC 1 and 2. **b** Volcano plot marked some potential target genes associated with cell adhesion and lipids metabolism. **c** KEGG analysis revealed specific features in the enriched pathway of two EC subtypes, in which EC 1 mainly enriched in atherosclerosis-related pathways. **d** Bar plot showing KEGG terms enriched in EC 1 in different segments, matched with the DEGs highly expressed in the AOAR and AA1 parts. The Y axis represents the ratio of upregulated genes to total DEGs in corresponding terms. Bar plot showing KEGG terms enriched in EC 2 in different segments, matched with the DEGs that were highly expressed in the AOAR segment, like PPAR signaling pathway. **e** Verification of *Vcam1* expression in ECs across five segments showed that the expression of *Vcam1* were enriched in the AOAR portion by immunofluorescent localization. Scale bar = 10 μm. **f** Negative control for *Scarb2* and *Vcam1* staining in the aorta. Scale bar = 10 μm. DEGs, different expression genes. Each experiment was repeated independently for a minimum three times.

**Fig. S4. The features of two fibroblast subtypes, in which co-localization of gene *Fbn1* with fibroblasts identified in fibroblast 1 and focused on thoracic segments.**

**a** Average expression and degree of top markers in fibroblast 1 and 2. **b** Violin plot provided supporting evidence of the most representative genes *Pi16* of fibroblast 1 and *Fmod* in type 2. **c** KEGG analysis of fibroblast subtypes manifested the pathways and biological functions related to the mechanism of inflammation reaction and extracellular matrix. **d** The Fbn1^+^ fibroblasts in the t-SNE plot matched with fibroblast 1. Violin plots of expression of *Fbn1* in two fibroblast subtypes manifested a significant high level in Fibro 1. The expression level of *Fbn1* in fibroblasts 1 was much higher in thoracic parts, particularly AOAR segment. E. The distribution of Fbn1^+^ fibroblast mostly concentrated on the AOAR segment and gradually decreased from AOAR to AA2 segments. **f** Negative control for *Fbn1* staining in the aorta. Scale bar = 10 μm.

**Fig. S5. Sca1^+^VSMC marked with *Ly6a* (*Sca1*) and KEGG analysis of all VSMC subtypes**

**a** The stem-like VSMCs marked with *Ly6a* are detected in the all VSMCs and matched with the cluster 10. **b** Each VSMC cluster plays its respective roles in the VSMC system, according to pathway analysis. VSMC 1 showed fewer enriched pathways compared with the other subtypes. **c** Positive control for *Malat1* in lung tissue and negative control for *Malat1* in situ hybridization by the probe target *dapB* in the aorta. Scale bar = 20 μm.

**Fig. S6. The rest marker genes of VSMC 5 and monocytes analyzed by GO analysis. a** VSMC 5 and monocytes appeared close location in terms of the t-SNE algorithm. **b** The classic marker gene *Cd14* was used to verify the identification of Monocytes. **c, d** Comparing the marker gene of VSMCs and Monocytes respectively by the expression ratio and the number of cells to identify VSMC 5 as VSMCs essentially. **e** The t-SNE and violin plot highlighted the key genes associated with the immune process and inflammation reaction. These genes of VSMC 5 demonstrated a similar profile but weaker expression level than the monocytes. **f** In some biological synthesis process, VSMC 5 might serve monocyte-like function in whole VSMC system. **g** The enriched pathways of VSMC 5 contained lysosome, chemokine signaling pathway, and other immune-related processes.

**Fig. S7.** **The evaluation of inhibiting *Malat1* protected aorta from AAA both *in vitro* and *in vivo*.**

**a** KEGG analysis showed down-regulated genes in Malat1^-/-^ MOVAS cells than in the WT type. **b, c** The statistical analysis of protein level of MMP2, MMP9, IL-1β, TNF-α in vivo by western blotting and immunohistochemistry. n=6 per group. **d** The grade of elastin degradation in mice with the injection of DMSO and MALAT1-IN-1 before Ang II infusion. **e** Representative computed tomography angiography (CTA) images of abdominal aortas from mice with the injection of DMSO and MALAT1-IN-1 in the last two weeks with continuous Ang II infusion. The diameter of abdominal aorta was significantly lesser by two weeks inhibitor injection showing its effect of arresting AAA progression. **f** Representative computed tomography angiography (CTA) images of abdominal aortas from mice with the injection of DMSO and MALAT1-IN-1 in the last two weeks after Ang II removing. The diameter of abdominal aorta was significantly lesser by two weeks inhibitor injection compared with both 2WA and 2WA+PBS group showing its reversing effect of AAA. **g, h** The grade of elastin degradation in mice with the injection of DMSO and MALAT1-IN-1 in last two weeks with continuous Ang II infusion and removing Ang II infusion separately. Student’s t test was used for **Fig. S7b, c.** Differences in the grading of AAA were analyzed with chi-squared test for **Fig. S7d, g, h**. *P<0.05, ** P<0.01, *** P < 0.001, **** P < 0.0001.

**Fig. S8. Cell to cell interaction analysis with WGCAN and Circos plots**

**a** WGCAN analysis of all cell subtypes manifests a similar gene module between them, particularly VSMCs 2, 3, 4. **b** The clustering of cell subtypes proves the close relationship between subtypes, even belonging to different types. **c** Cell-to-cell interaction provides the cellular network and control center of all cell subtypes. **d** The specific genes interaction among all cell types and all the VSMCs selected separately with Circos plots.


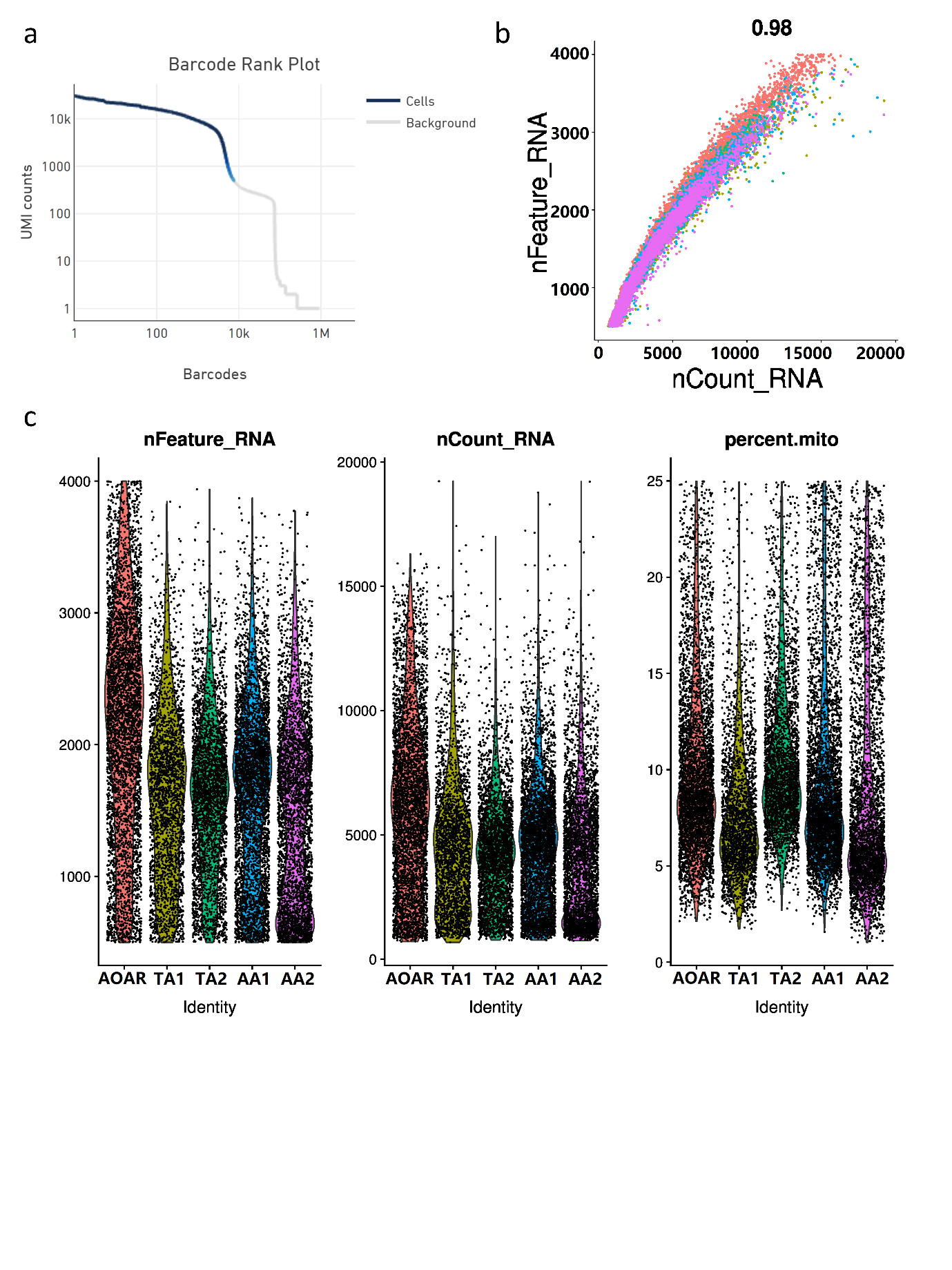


Figure. S1. Quality Control (QC) plots.
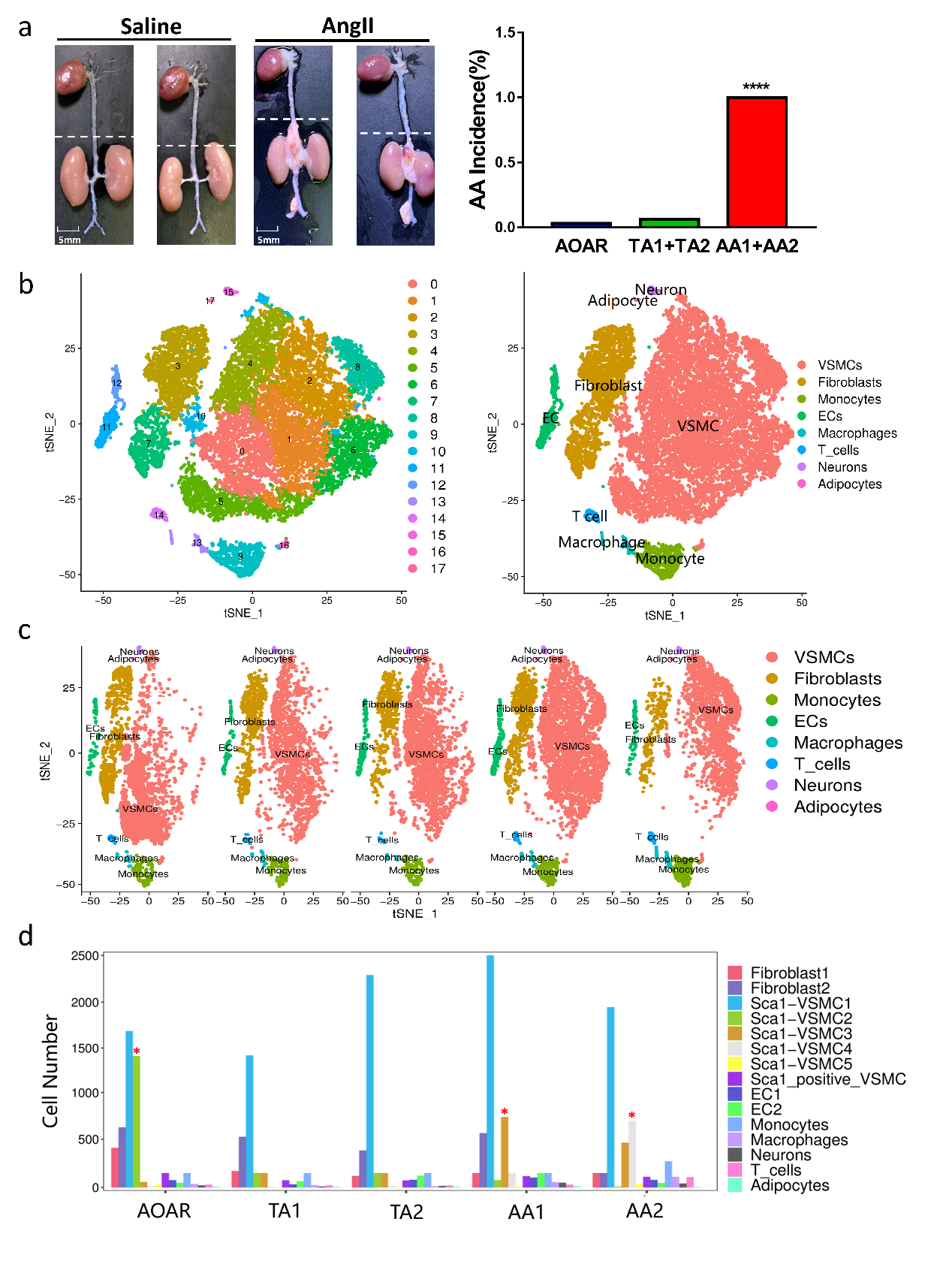


Figure. S2. Ang II induced AAA mice model and cell composition of all aortic segments.


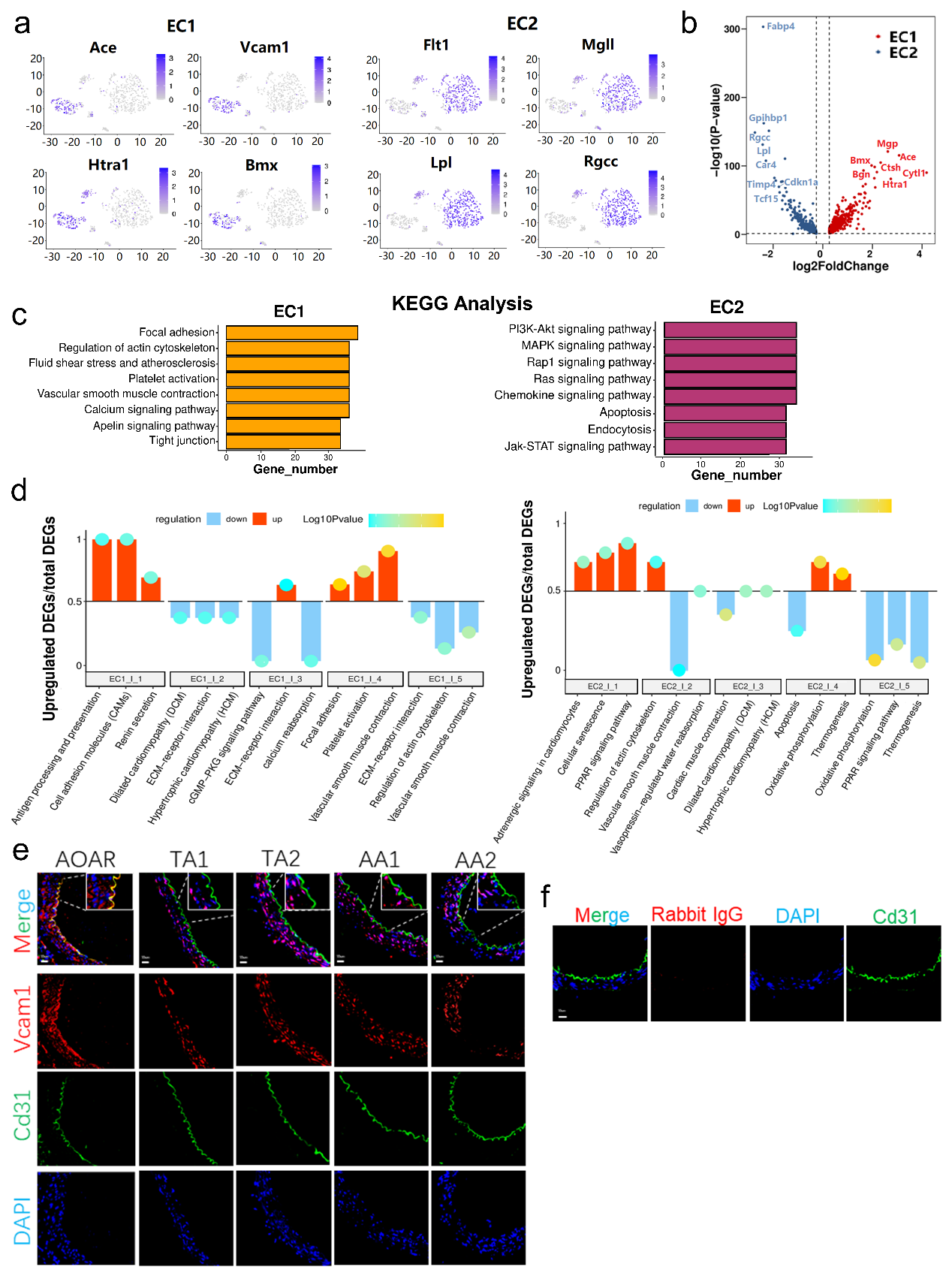


Figure. S3. The marker genes identified ECs subtypes and related KEGG analysis.


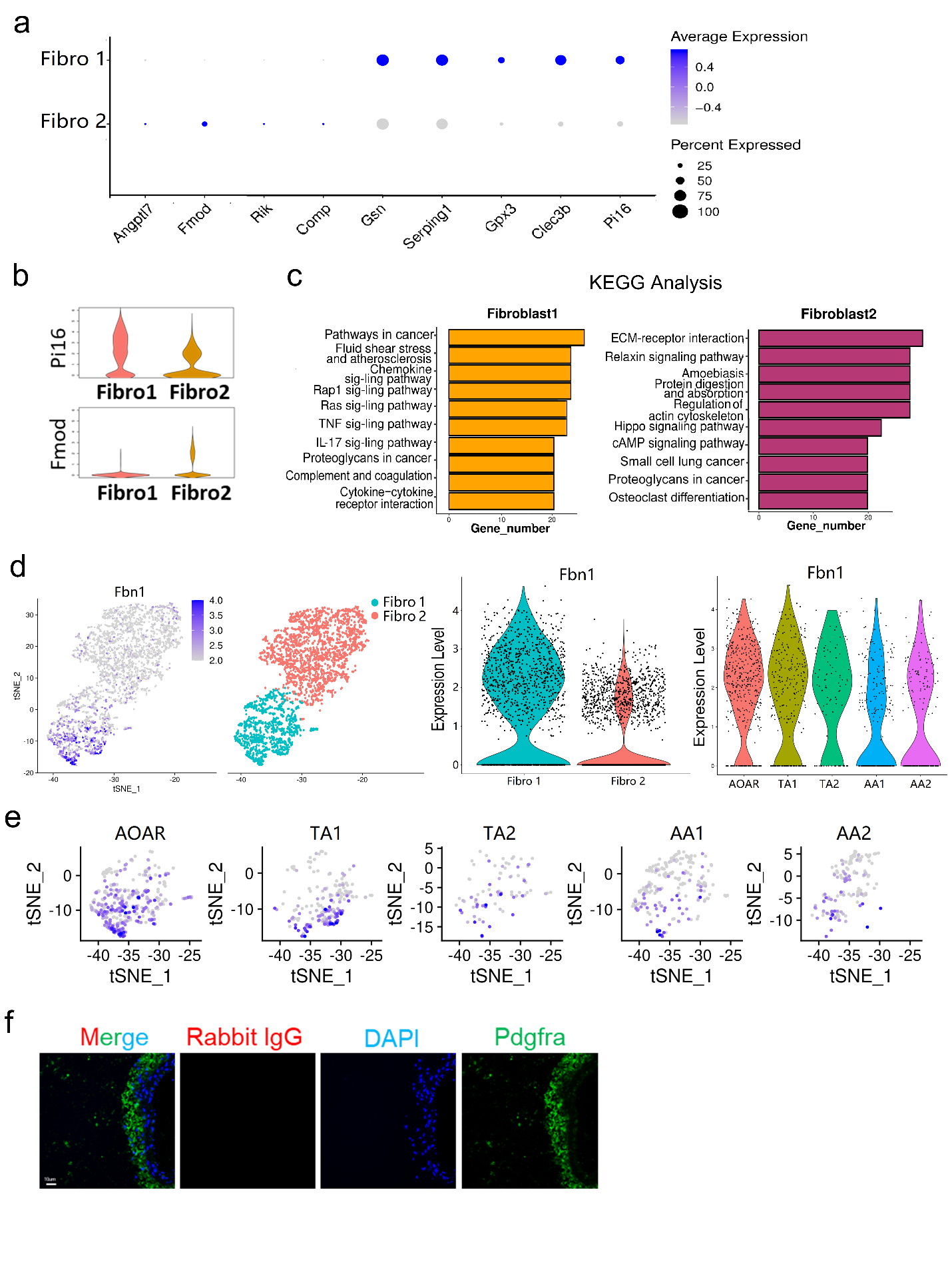
**Figure. S4. The features of two fibroblast subtypes, in which co-localization of gene Fbn1 with fibroblasts identified in fibroblast 1 and focused on thoracic segments.**


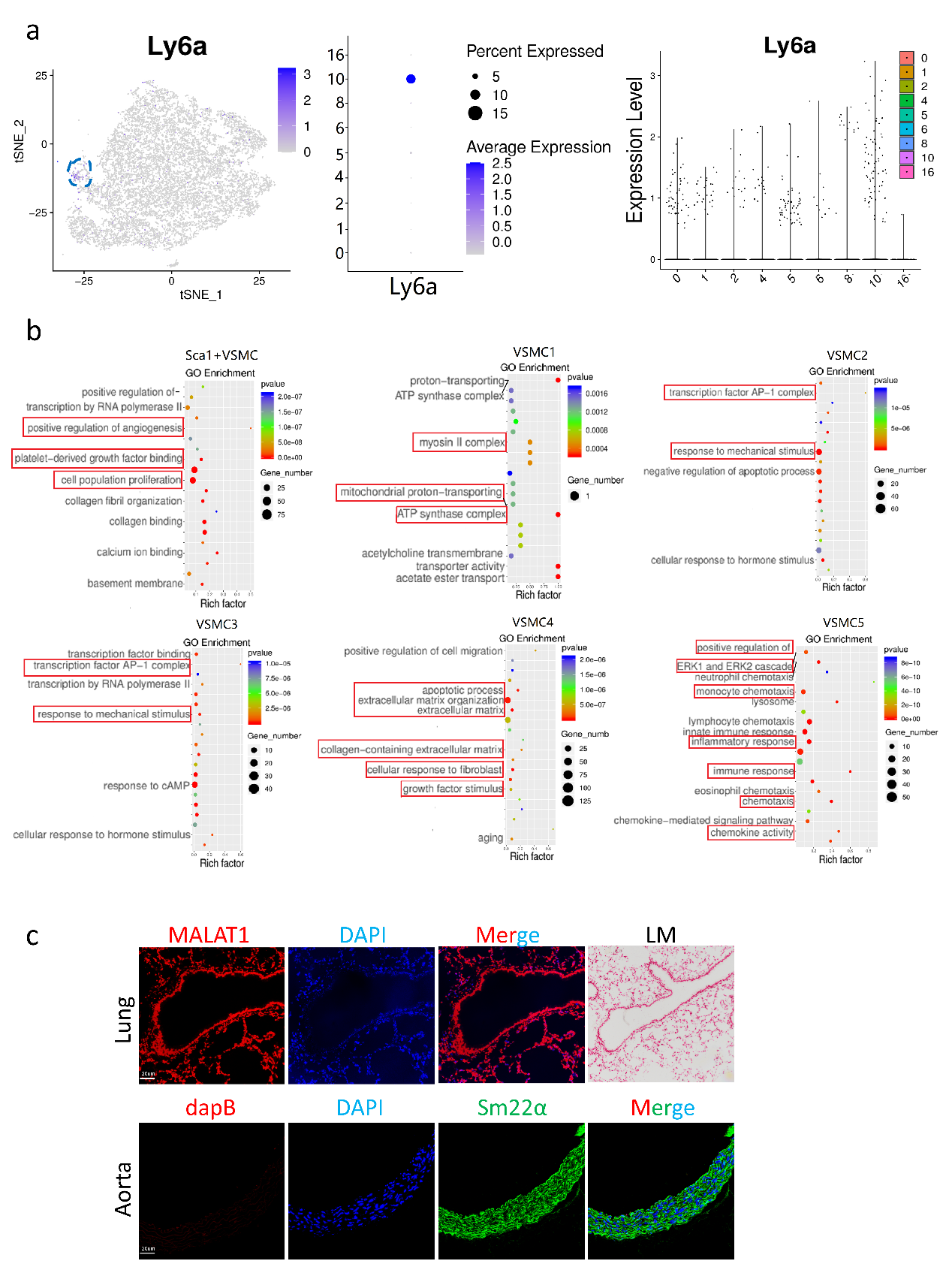


Figure. S5. Sca1+VSMC marked with Ly6a (Sca1) and KEGG analysis of all VSMC subtypes.


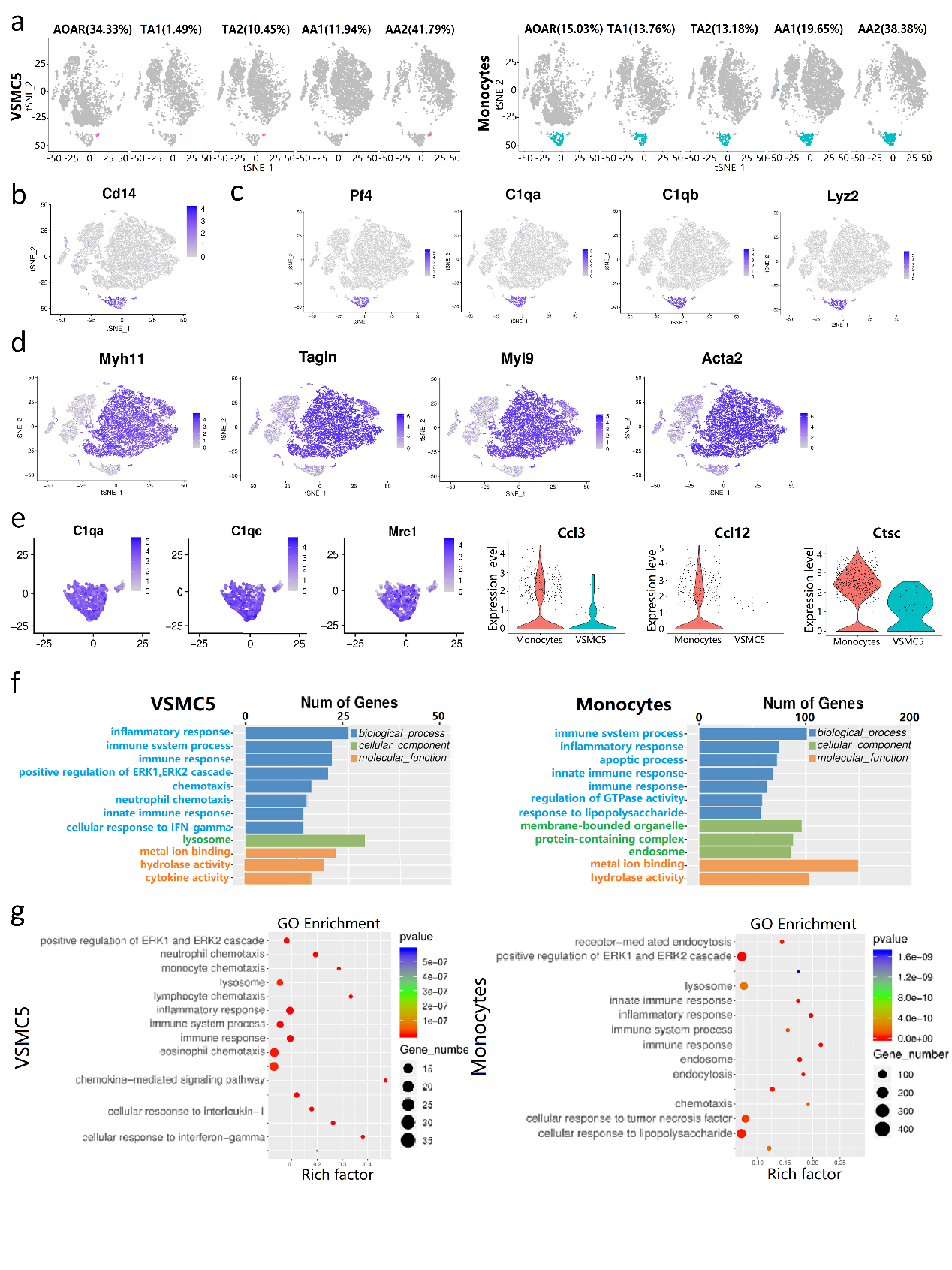


Figure. S6. The rest marker genes of VSMC 5 and monocytes analyzed by GO analysis.


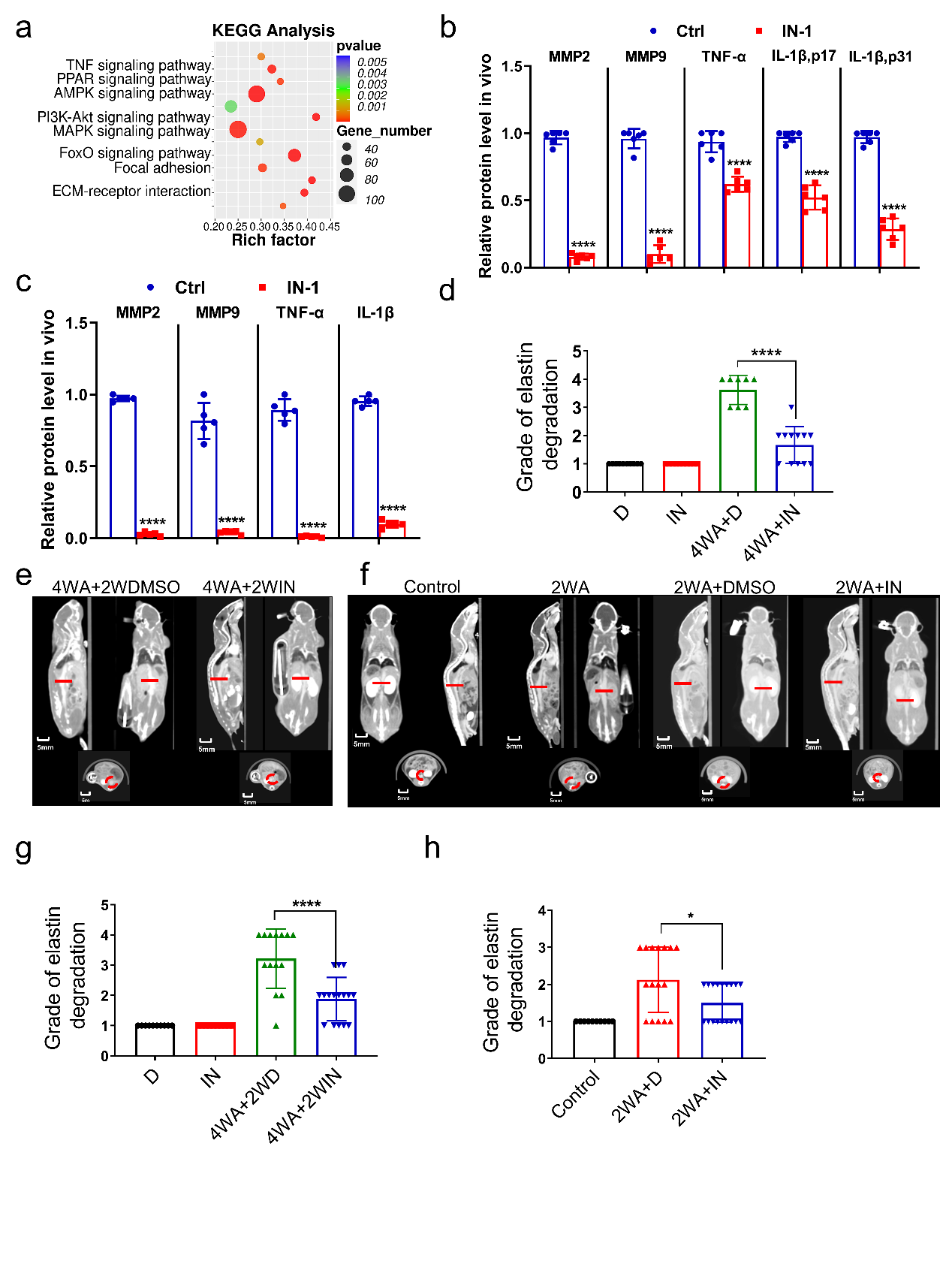


Figure. S7. The evaluation of inhibiting Malat1 protected aorta from AAA both in vitro and in vivo.


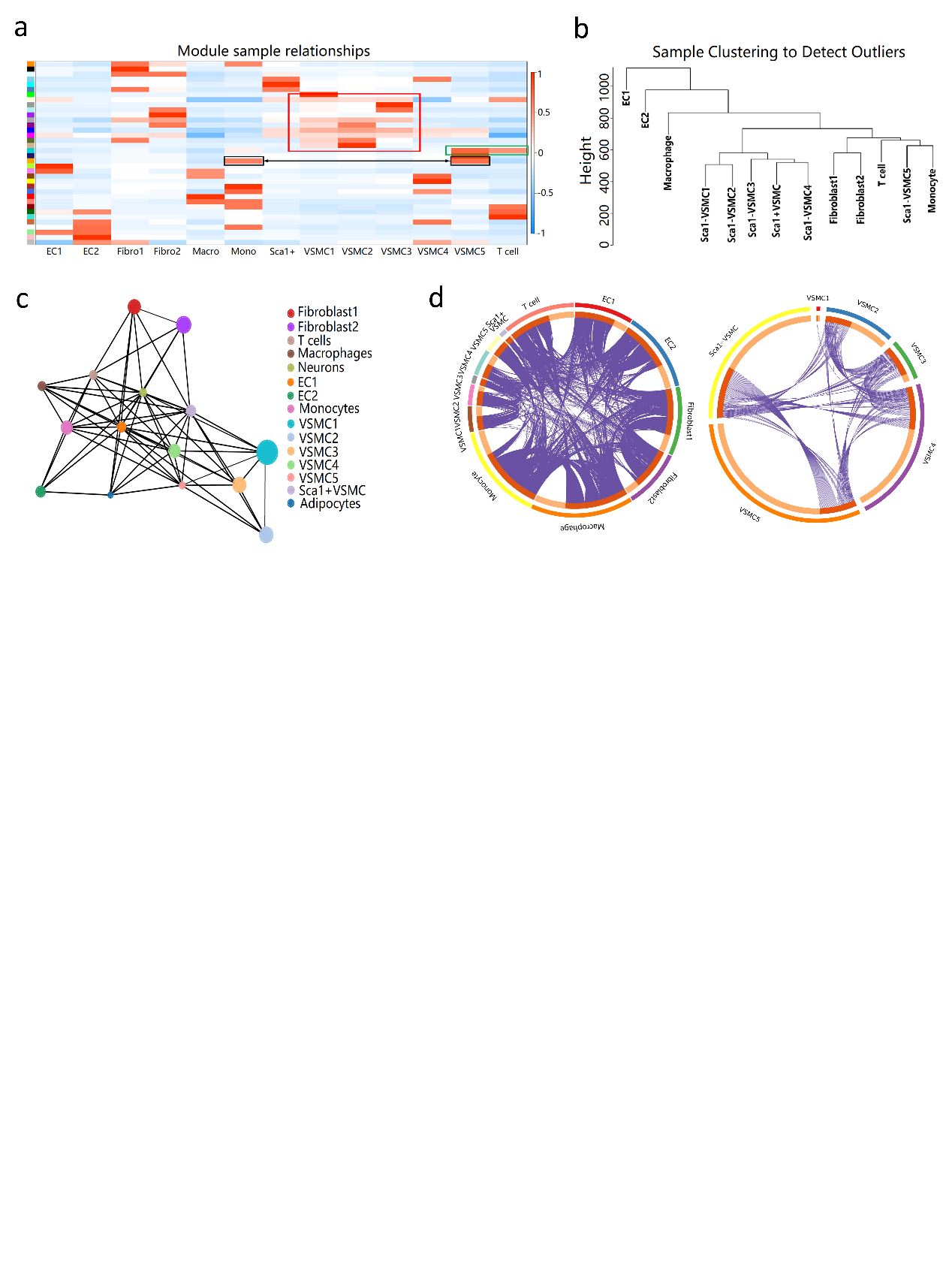


Figure. S8. Cell to cell interaction analysis with WGCAN and Circos plots.

| **Gene name** | **Sequence 5’-3’** |
| --- | --- |
| mouse *β-actin* forward | GGCTGTATTCCCCTCCATCG |
| mouse *β-actin* reverse | CCAGTTGGTAACAATGCCATGT |
| mouse *mmp2* forward | CAGGGCACCTCCTACAACAG |
| mouse mmp*2* reverse | CAGTGGACATAGCGGTCTCG |
| mouse *mmp9* forward | TAGATCATTCCAGCGTGCCG |
| mouse *mmp9* reverse | GCTTAGAGCCACGACCATACA |
| mouse *tnf-α* forward | AGCCGATGGGTTGTACCTTG |
| mouse *tnf-α r*everse | ATAGCAAATCGGCTGACGGT |
| mouse *il1β* forward | ACCTTCCAGGATGAGGACATGA |
| mouse *il1β* reverse | AACGTCACACACCAGCAGGTTA |
| mouse malat1 forward | ATAGCCCAGGAAAGAGTGCG |
| mouse malat1 reverse | GCTTCACCACCACATCCGTA |
|  |  |
| **SgRNA sequence** |  |
| Sg2 | GGCCTTGTAGATTAAAACGA |
| Sg6 | GAGTCAAGCAAAGACACCGC |

Table S1. Prime sequence of genes


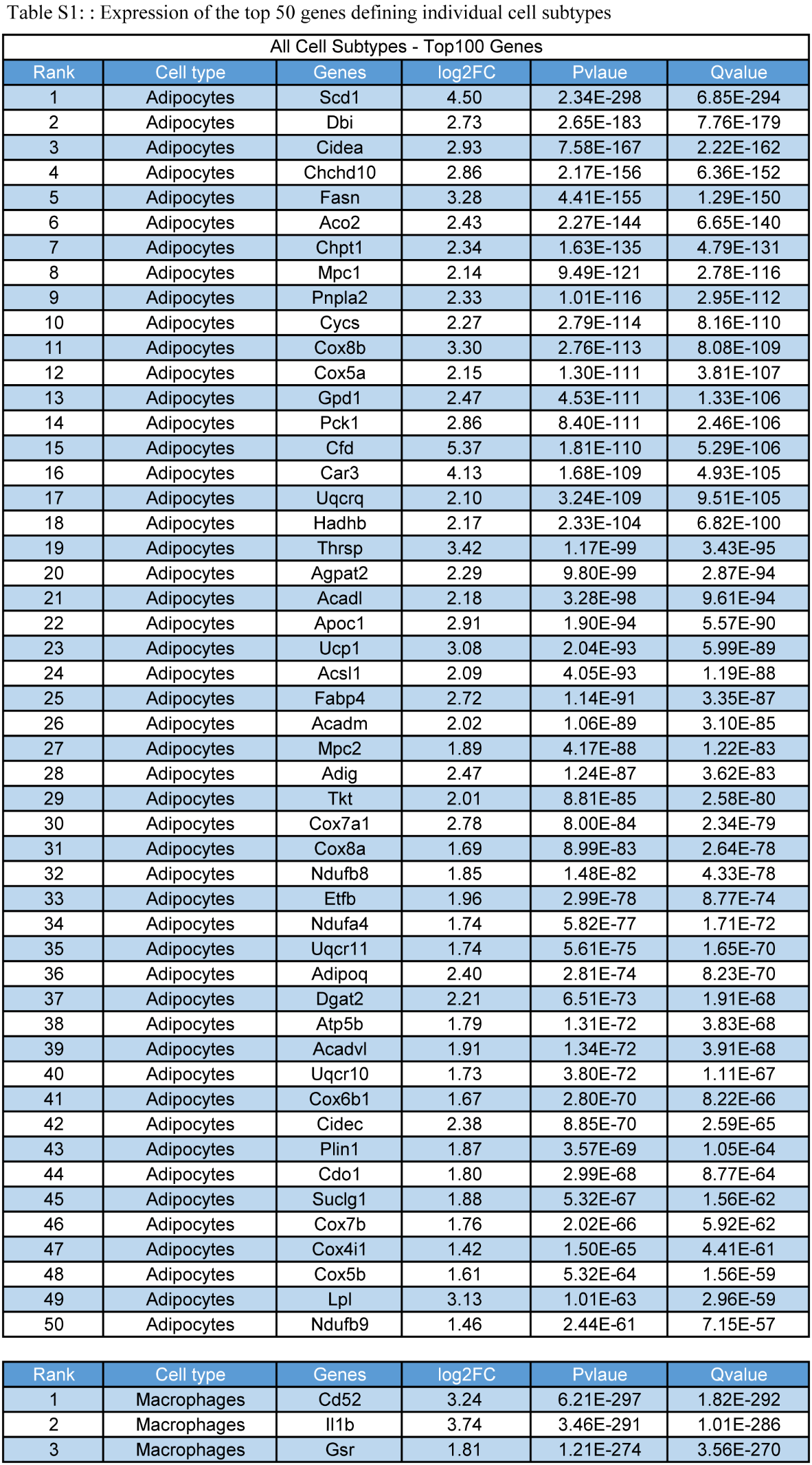


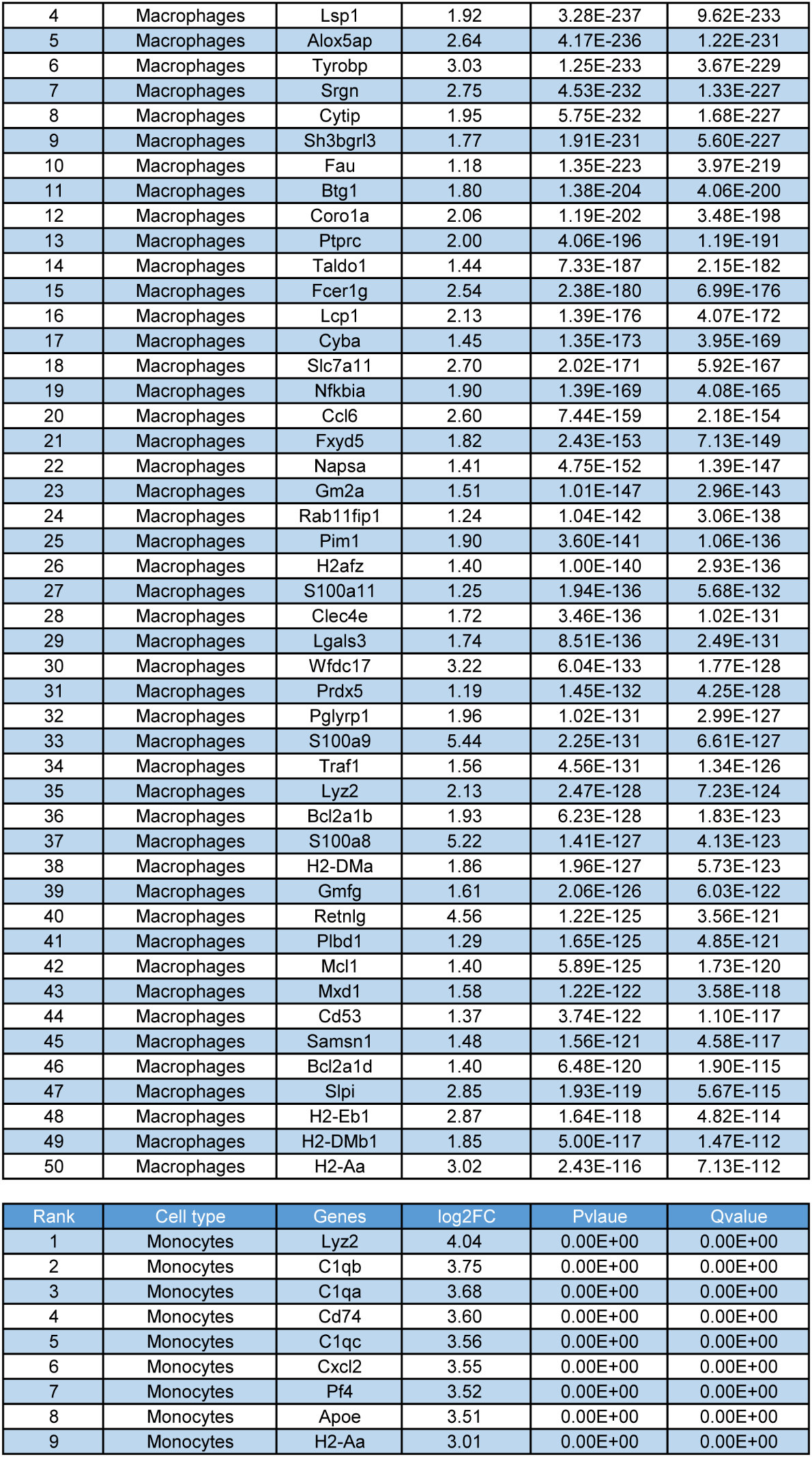

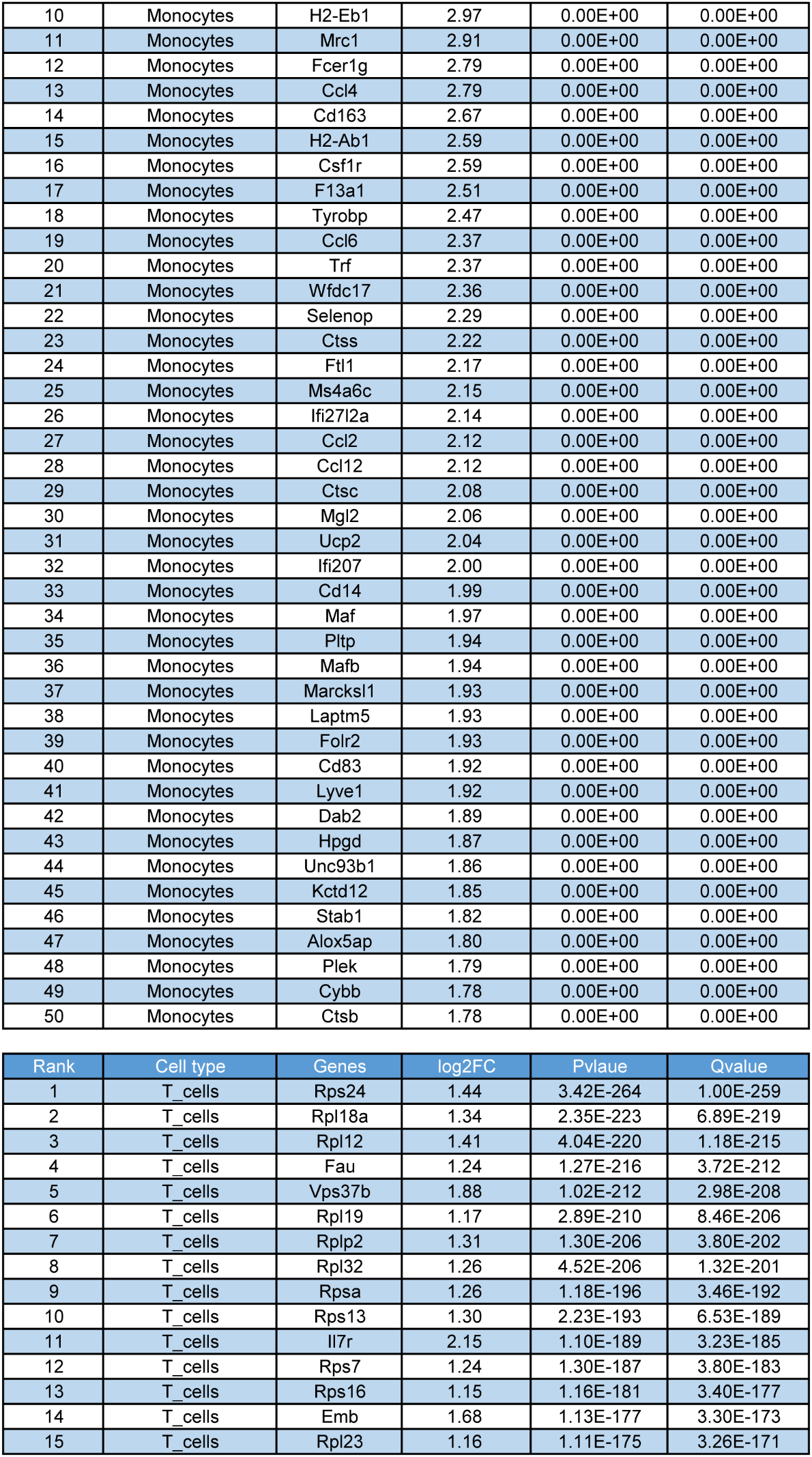

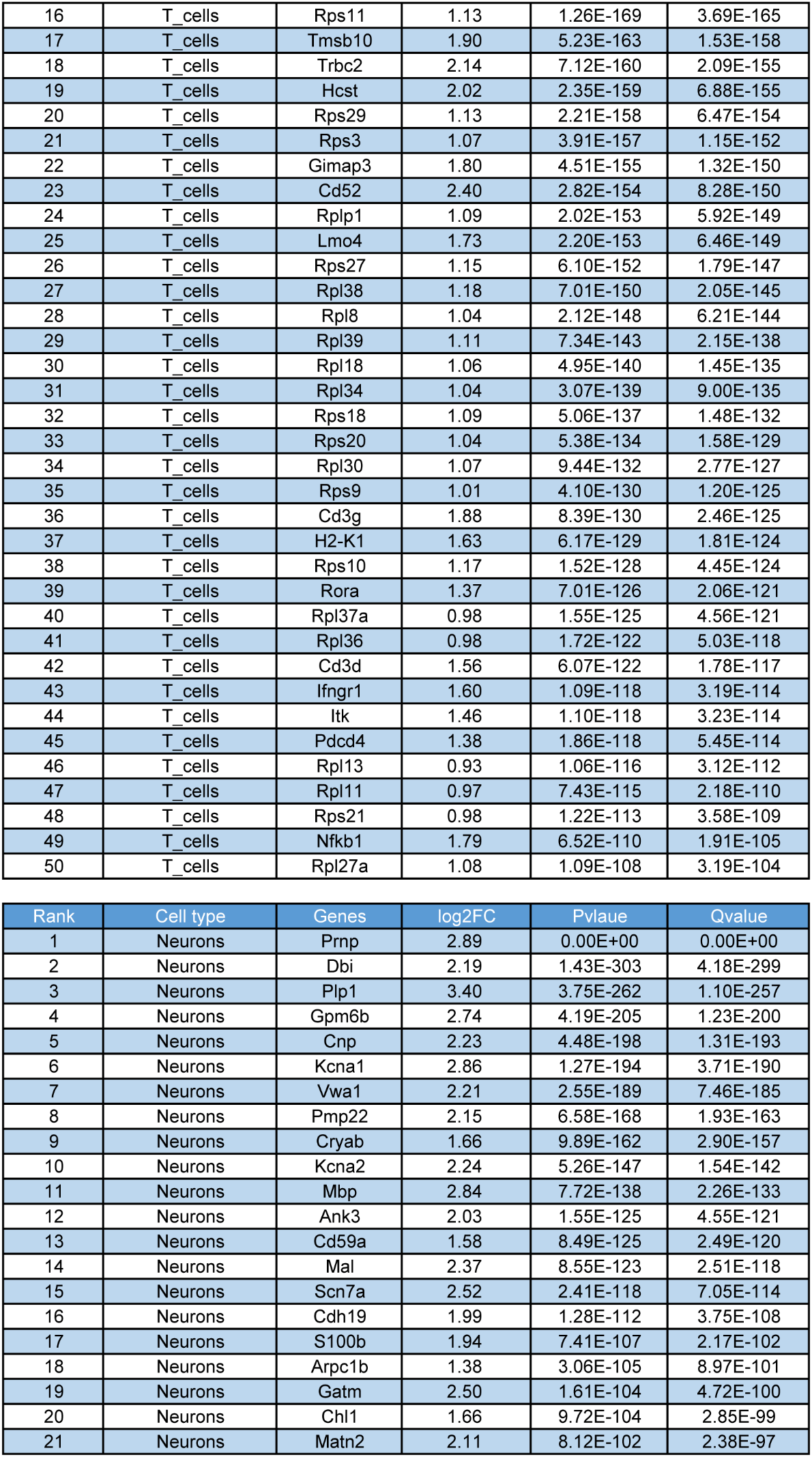

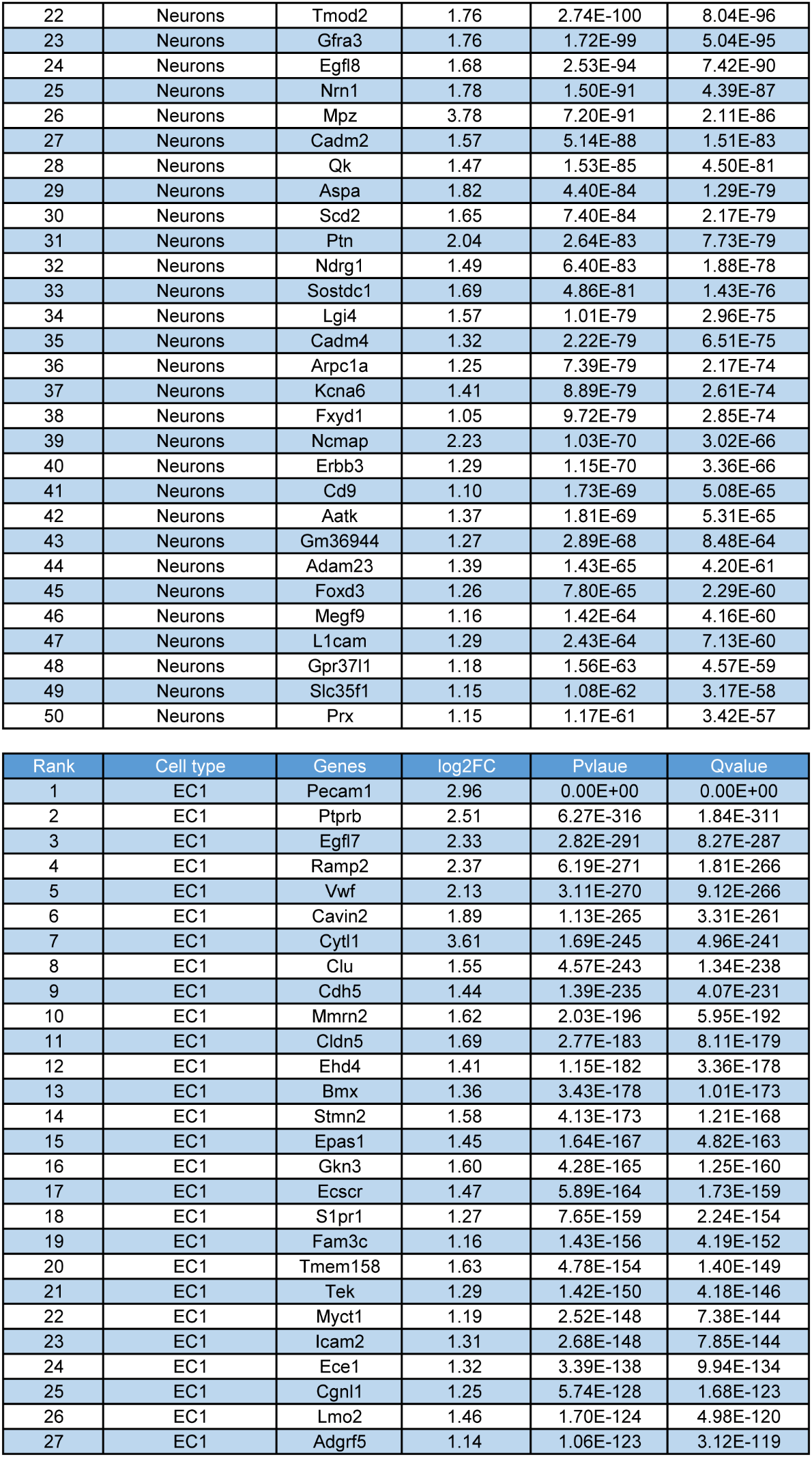

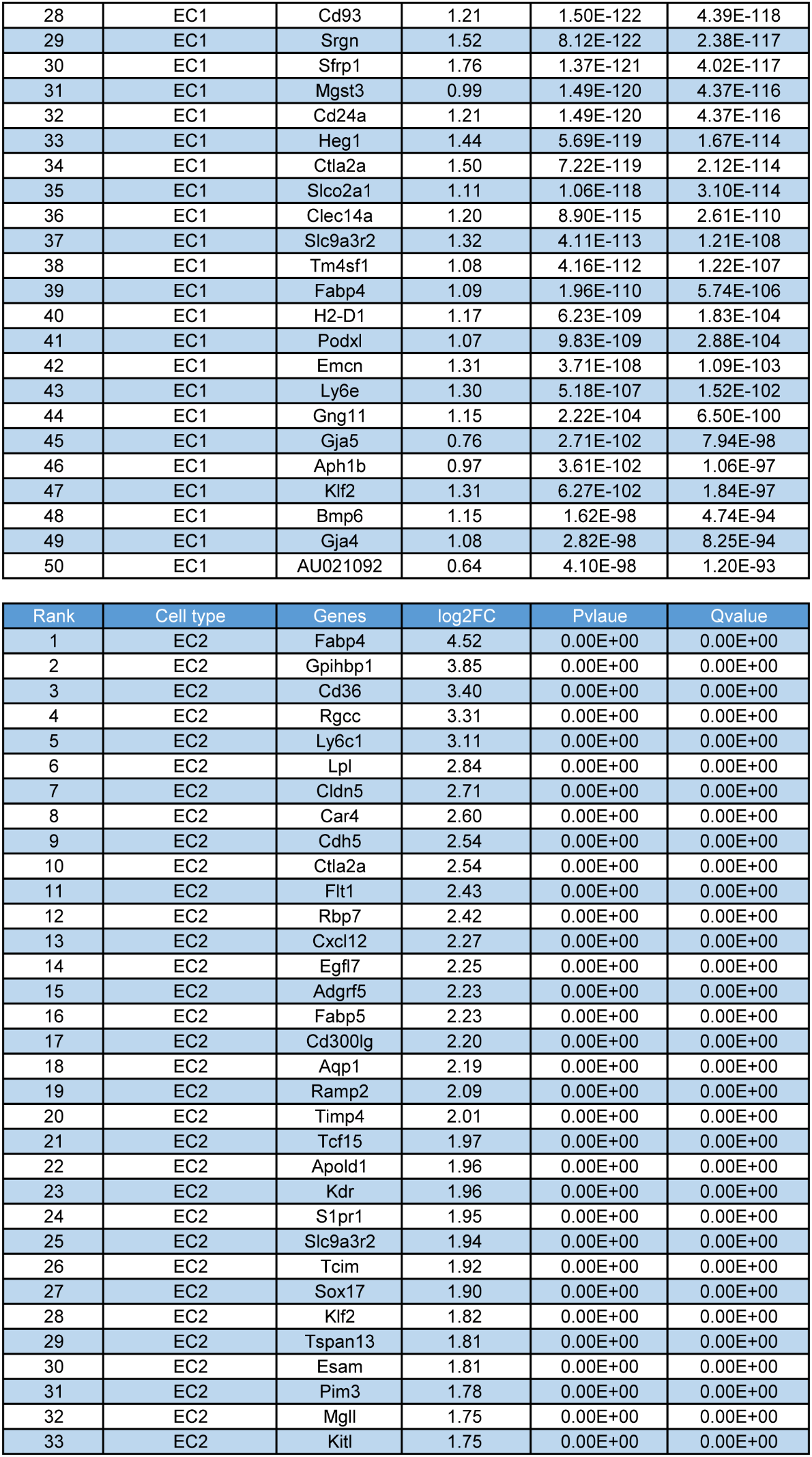

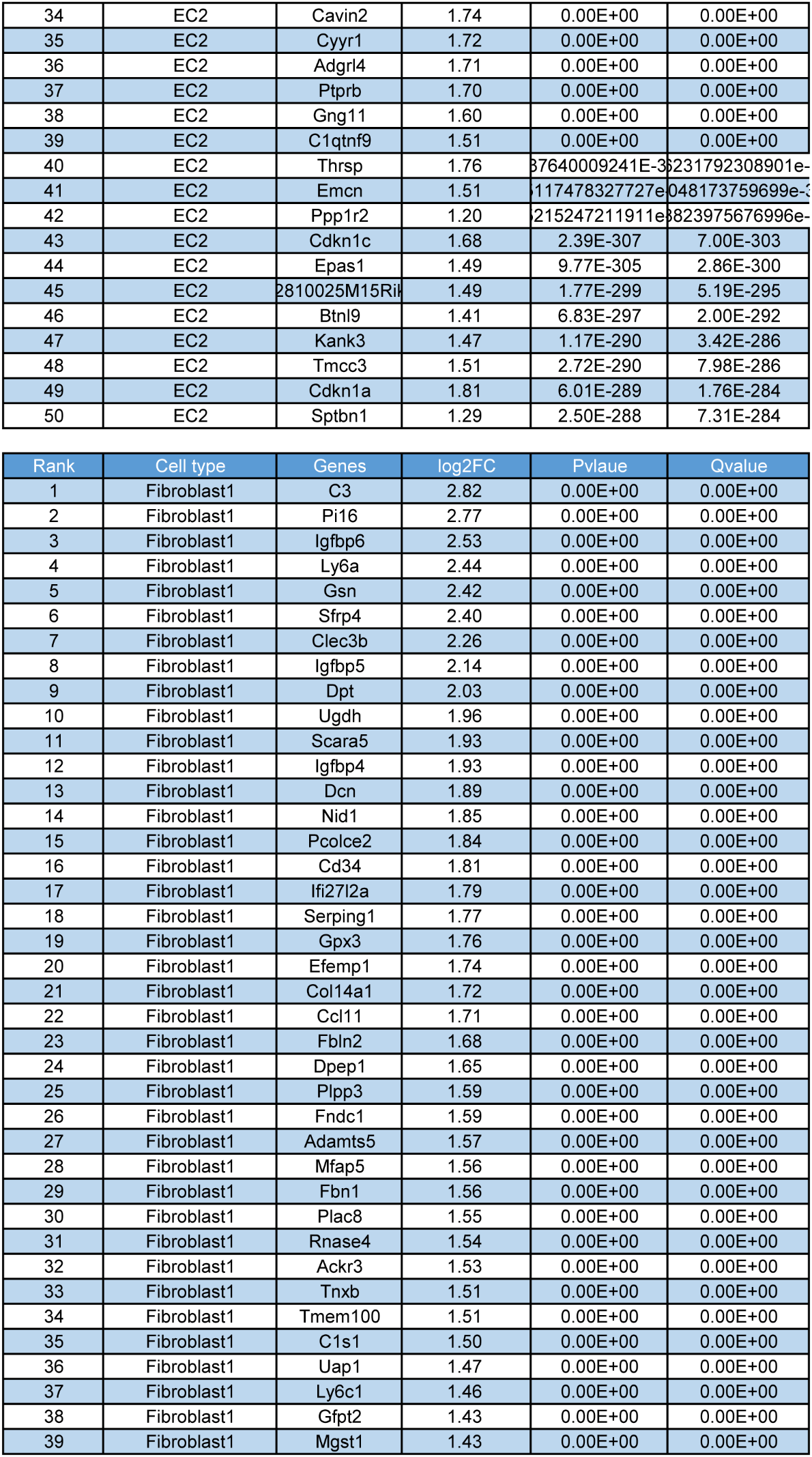

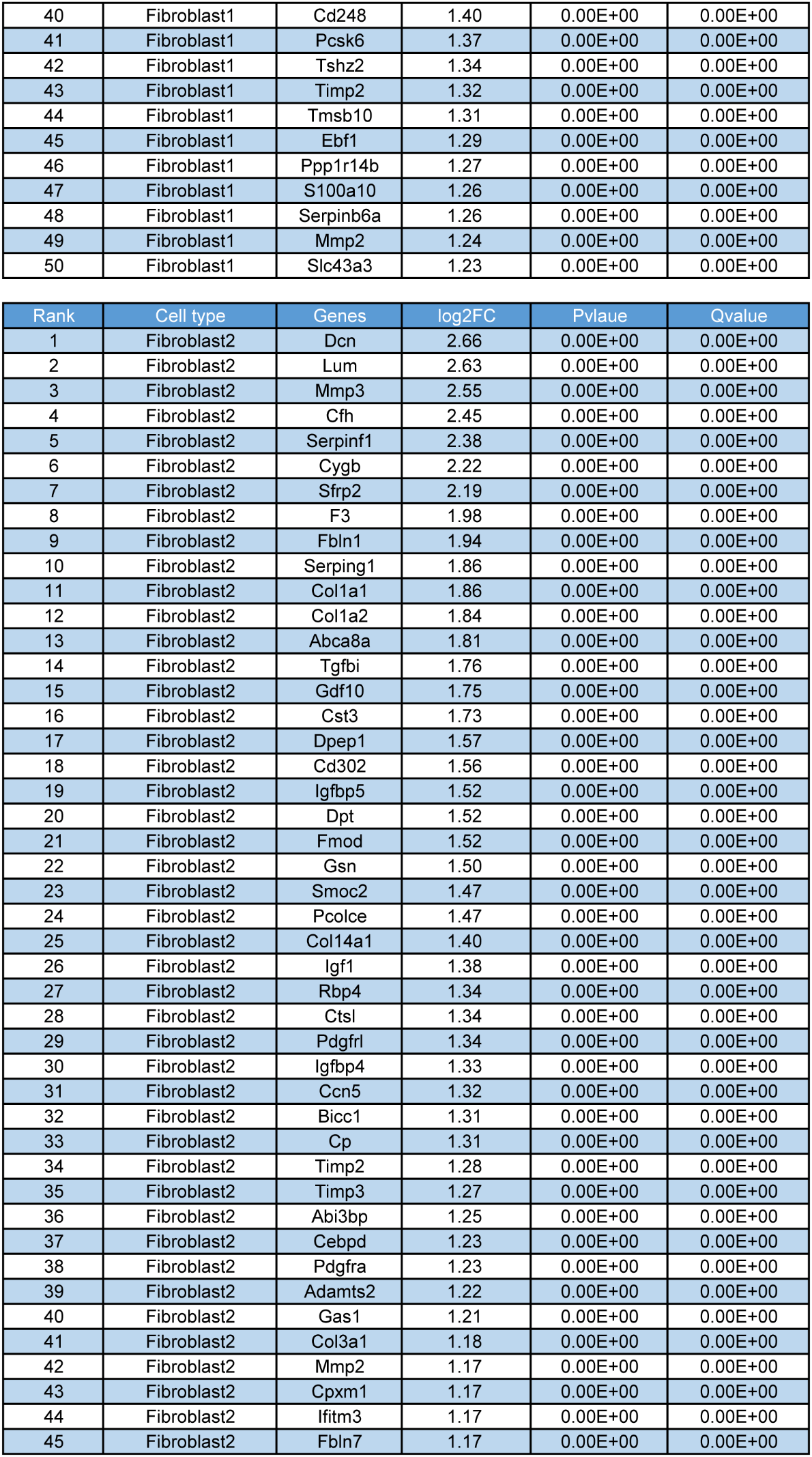

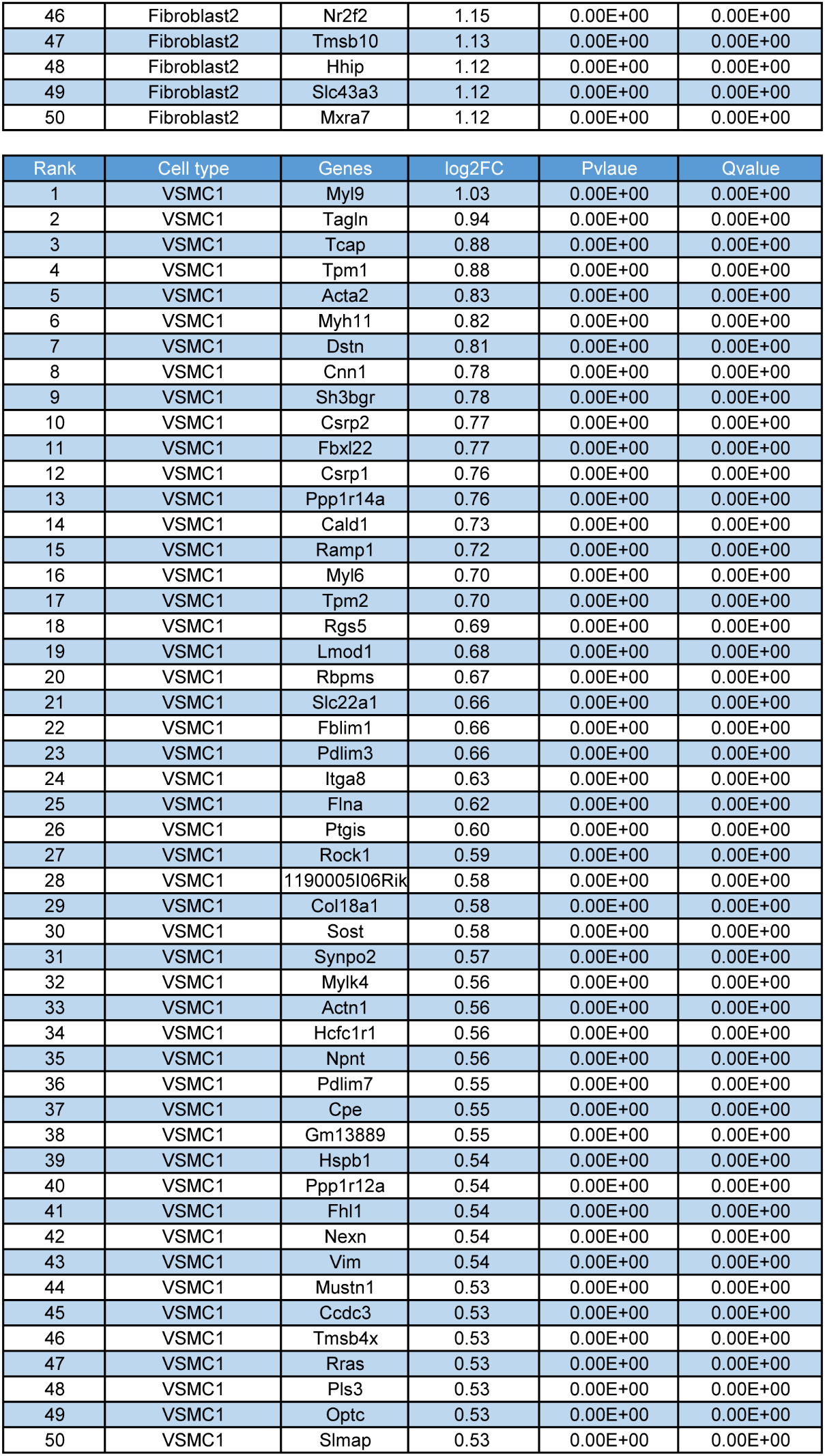

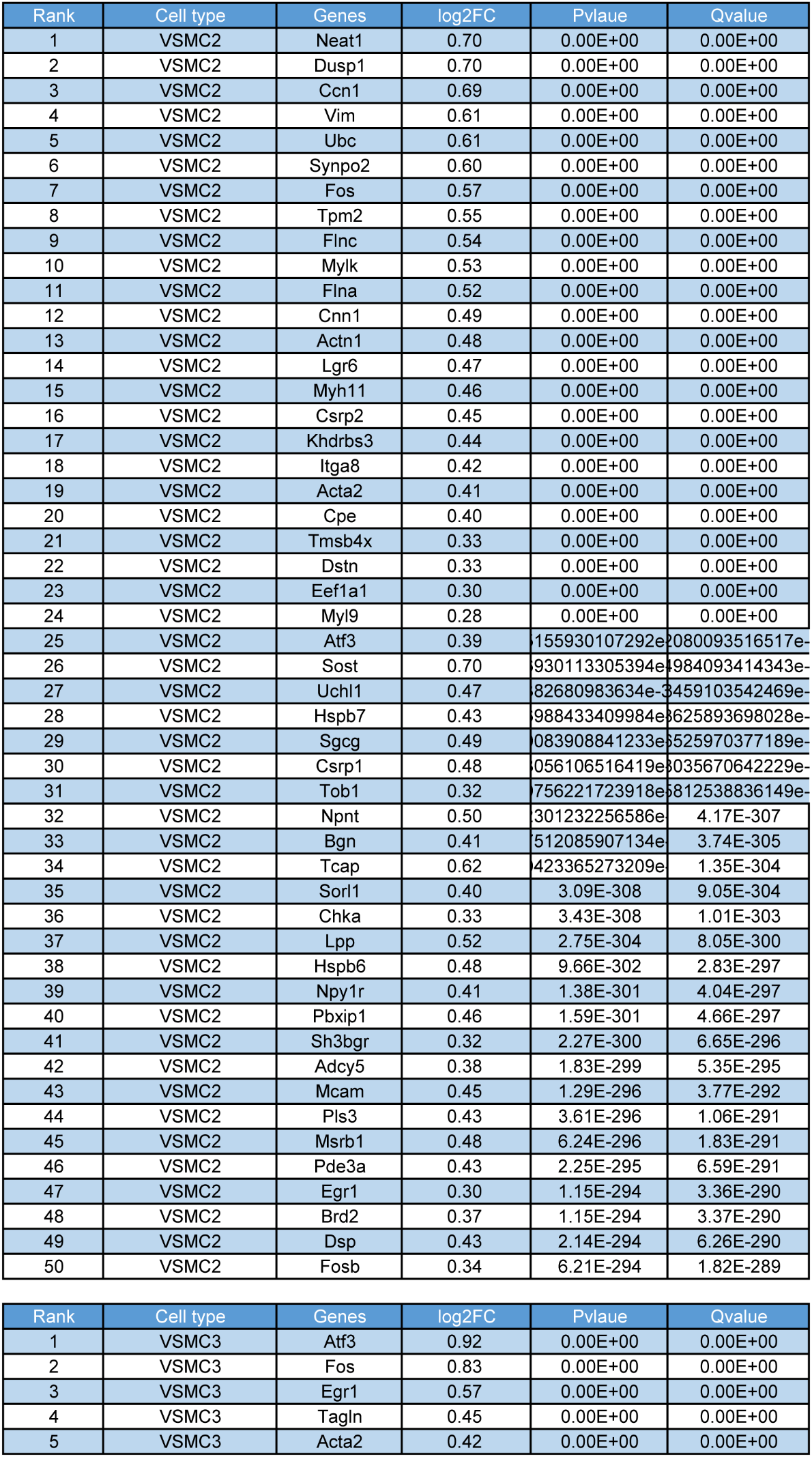

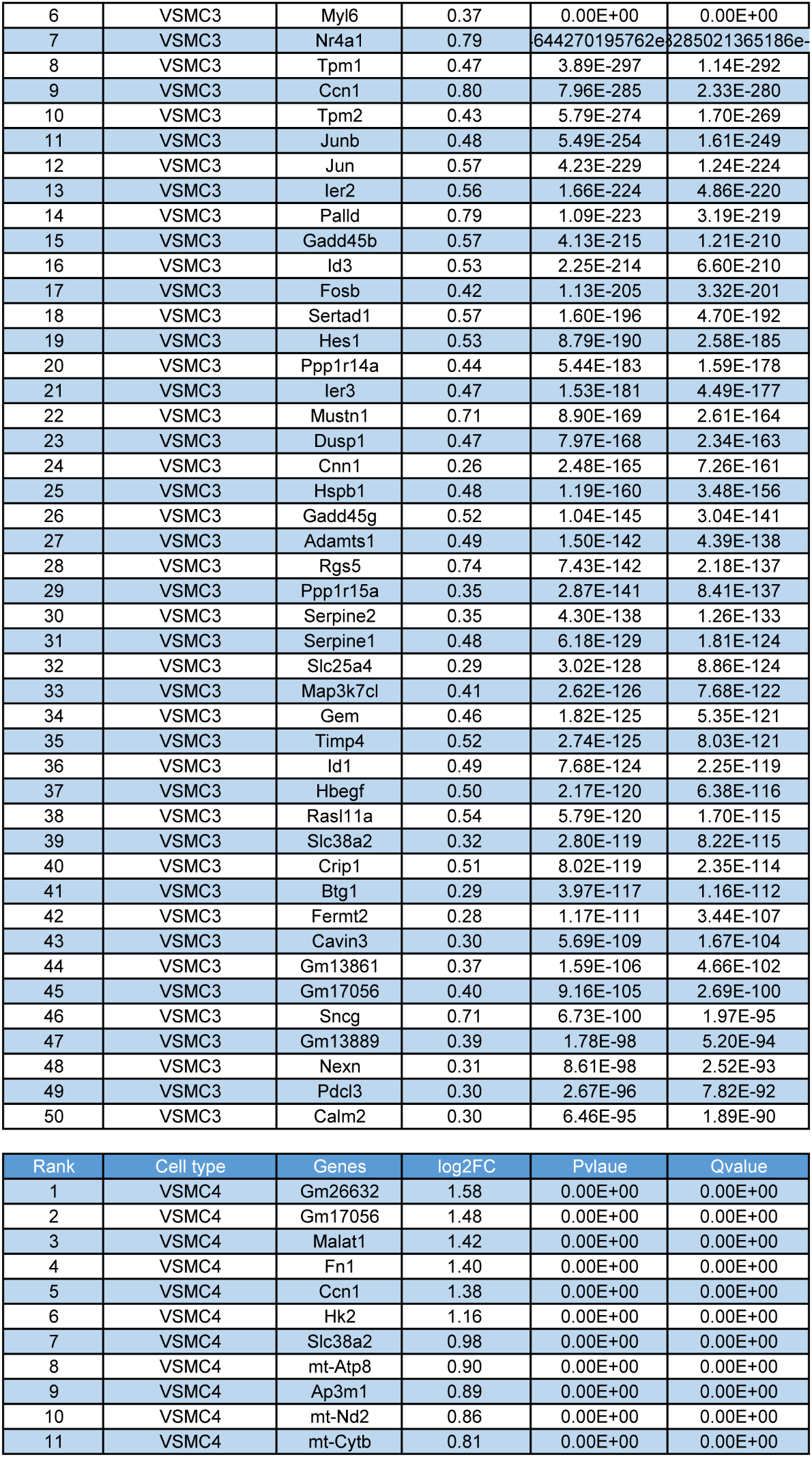

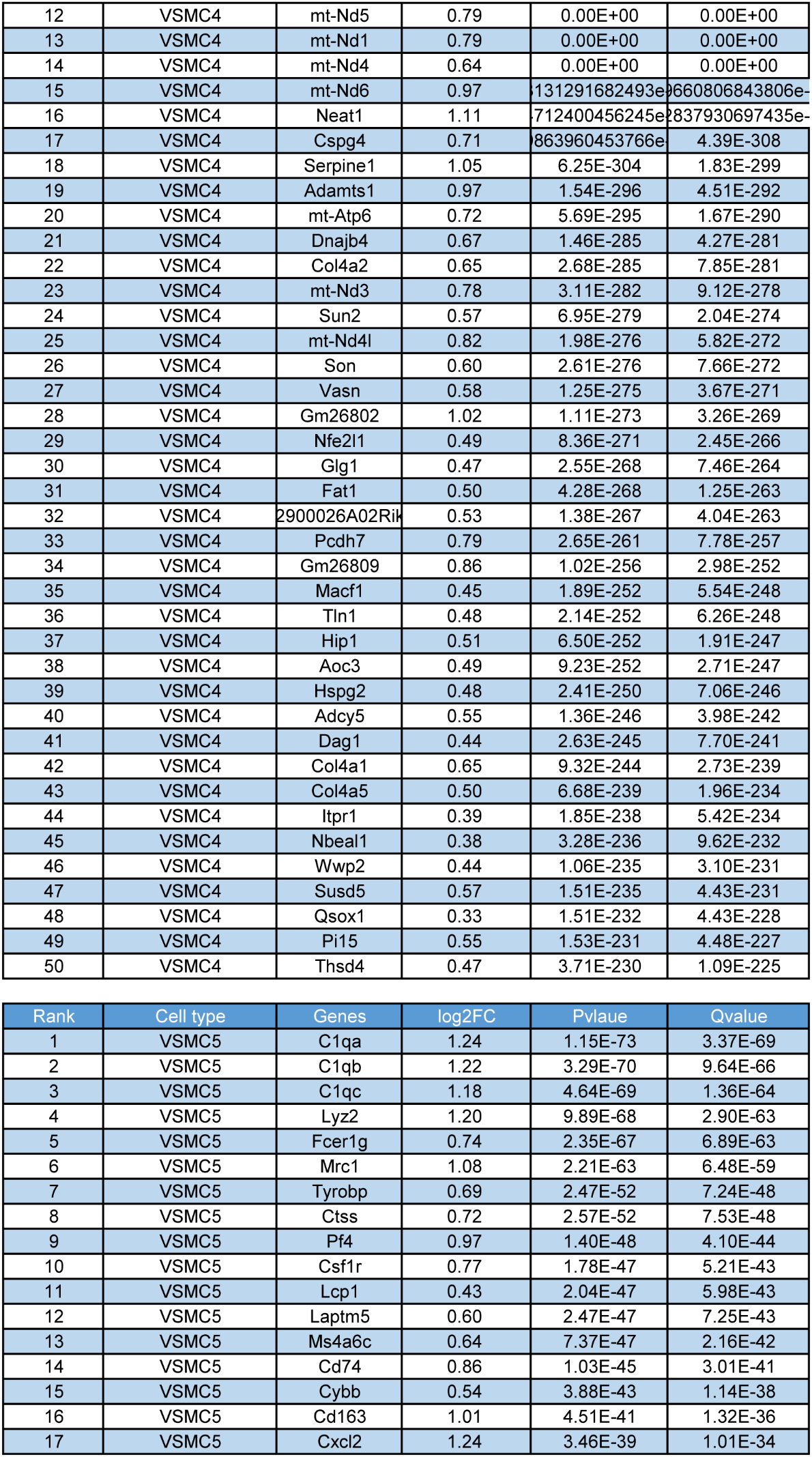

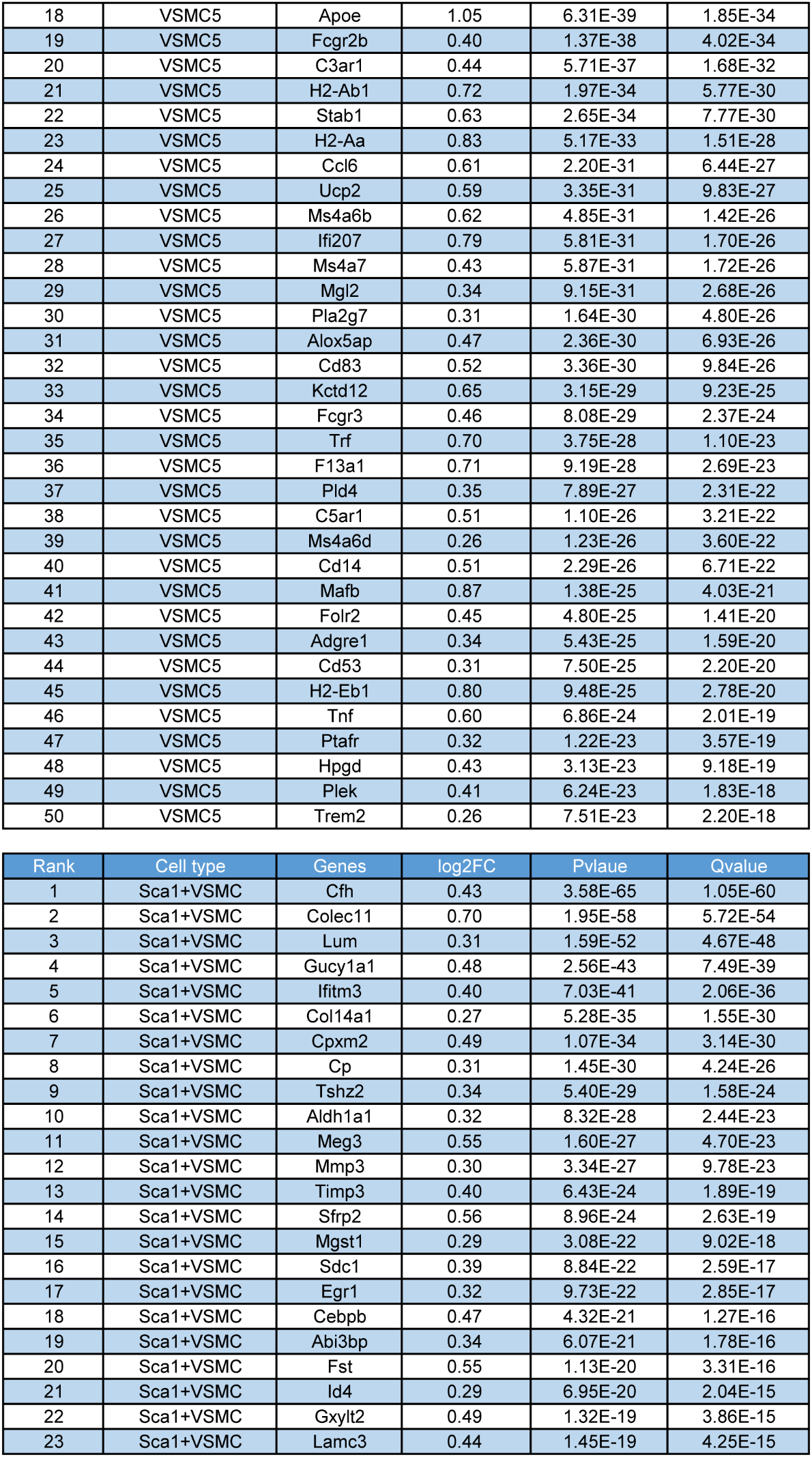

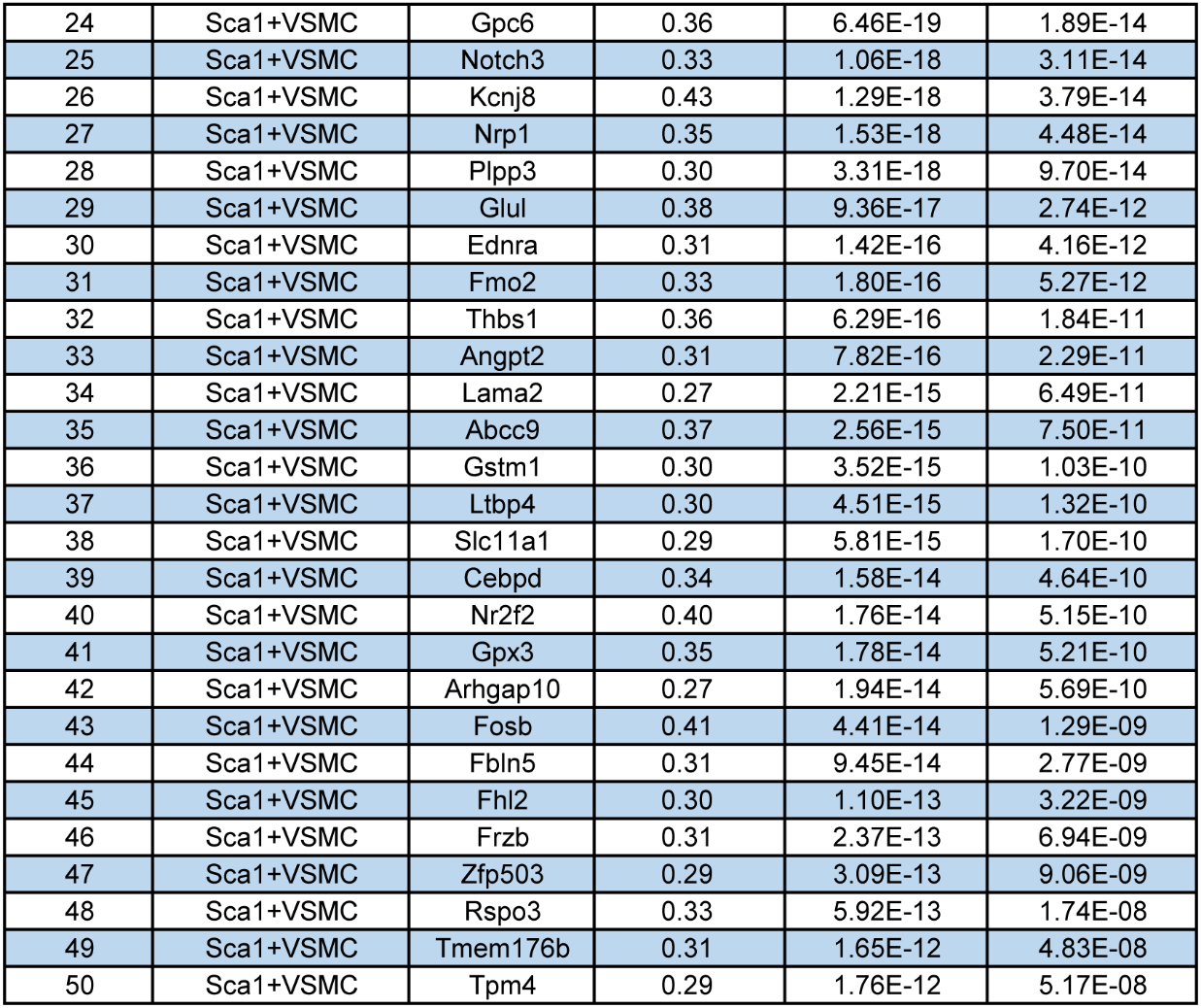


Table S2. Expression of the top 50 genes defining individual cell subtypes

Sca1^+^VSMC

VSMC1

VSMC2

VSMC3

VSMC4

VSMC5

Table S3. The enrichment pathways of VSMC subtypes

Table S4. The expression pattern of top DE genes found in Malat1 KO experiment
